# scDETECT: a novel statistical model accounting for cell type correlation in single-cell RNA-seq differential expression analysis

**DOI:** 10.1093/bib/bbaf556

**Published:** 2025-10-27

**Authors:** Yuhan Xu, Weiwei Zhang, Hao Wu

**Affiliations:** Faculty of Computer Science and Control Engineering, Shenzhen University of Advanced Technology, No. 1068 Xueyuan Avenue, Nanshan District, Shenzhen, Guangdong 518055, China; School of Information Engineering, Jingdezhen Ceramic University, No. 883 Xihu Avenue, Changjiang District, Jingdezhen, Jiangxi 333403, China; School of Mathematics Information, Shaoxing University, No. 508 Huancheng West Road, Yuecheng District, Shaoxing, Zhejiang 312000, China; Faculty of Computer Science and Control Engineering, Shenzhen University of Advanced Technology, No. 1068 Xueyuan Avenue, Nanshan District, Shenzhen, Guangdong 518055, China; Institute of Advanced Computing and Digital Engineering, Shenzhen Institute of Advanced Technology, No. 1068, Xueyuan Avenue, Nanshan District, Shenzhen, Guangdong 518055, China

**Keywords:** differential expression, single-cell RNA-seq, cell type correlation, Bayesian hierarchical model

## Abstract

Differential expression (DE) is one of the most important analyses in single-cell RNA-seq (scRNA-seq). Due to similarity of cell types, the DE states often have strong correlation among different cell types. Existing methods perform DE analysis for each cell type separately and ignore such correlation, leading to low accuracy, and statistical power. We develop *s*ingle *c*ell *D*ifferential *E*xpression *TE*st with *C*ell *T*ype correlation (scDETECT), a novel statistical method, for scRNA-seq DE analysis accounting for the cell type correlations. scDETECT implements a Bayesian hierarchical model to incorporate the cell type correlations into the modeling of the gene expression, and then the DE genes are called based on the derived posterior probabilities. Simulation and real data studies show that scDETECT significantly improves the accuracy and statistical power compared with existing methods.

## Introduction

Differential expression (DE) is the most fundamental analysis for transcriptomic data. Its main goal is to identify genes with expression levels associated with the outcome, such as distinct biological or clinical conditions. The research for DE analysis provides valuable insights into many important problems, e.g. the pathogenesis of diseases. Researchers can identify potential therapeutic targets through detecting DE genes related to diseases, thereby providing scientific guidance for drug development and precision medicine.

The methodological development for DE analysis has a long history, dating back to the gene expression microarray era [1]. With the development of the sequencing technology, there are many DE methods developed for bulk and single-cell RNA-seq (scRNA-seq). For bulk data, there are edgeR [2, 3], DESeq2 [4], BBSeq [5], DSS [6], baySeq [7], and ShrinkBayes [8, 9]. A common approach in these methods is to model the gene read counts by negative binomial (NB) distribution. The DE analysis is also an important question in scRNA-seq. Compared with bulk data, analysis on single-cell data can reveal cell-specific alteration, providing higher resolution compared with the bulk data. For scRNA-seq DE analysis, there are also many existing methods. Ren *et al*. [9] performed t-test between disease and normal group, while Tang *et al*. [10] conducted Wilcox test. Heterogeneity and multimodality, large amounts of zero counts, and sparsity of scRNA-seq data pose new challenges to the differential gene expression analysis. To solve these problems, Finak *et al*. [11] developed MAST, a two-part hurdle model, that employed logistic regression for expression rate and Gaussian model for expression level modeling, to handle sparse and bimodal single-cell data. There are also two extended forms of MAST, one is batch effect correction (MAST ComBat), and the other is individual random effect model (MAST RE) [12]. Single cell differential expression (SCDE) [13] also uses a two-part joint model for detecting differentially expressed (DE) genes to accommodate multimodal expression values and drop-out events. One part corresponds to the normal observed genes, the other corresponds to the drop-out events. Desingle [14] employs Zero-Inflated Negative Binomial (ZINB) regression model to estimate the proportion of real and dropout zeros and to define and detect three types of DE genes in scRNA-seq data with higher accuracy. scDD [15] considers four different modality scenarios for gene expression value distributions within and across biological conditions. SigEMD [16] combines a data imputation approach, a logistic regression model, and a nonparametric method based on the Earth Mover’s Distance to precisely and efficiently identify DE genes in scRNAseq data. scDD and SCDE are capable of detecting more subtle and complex differences in gene expression distributions than traditional methods that focus solely on mean shifts. Desingle and SigEMD show a good balance between sensitivity and accuracy in detecting DE genes. IDEAS [17] captures the cell type-specific gene expression of an individual by a probability distribution and then compare such distributions across individuals. SCDE is robust against noise due to its Bayesian error models but can be computationally intensive and assumes specific distributions that may not fit all data. Desingle excels in detecting DE genes with dropout-aware models, but it is also prone to false positives in noisy data. scDD has higher power to detect subtle differences in gene expression distributions and is able to characterize those differences, but it may struggle with datasets lacking clear modes. SigEMD is unique in leveraging earth mover’s distance to precisely and efficiently identify DE genes in scRNAseq data, but its computational complexity can be a limitation for large datasets.

Compared with the bulk data, the distinction of scRNA-seq DE analysis is that it is usually performed after cell clustering and cell type annotation, then the DE is performed separately for each cell type. However, the cell types often exhibit similarities, not only in their baseline expression, but also in their DE states. For example, Ji *et al*. [18] reported significant correlation among CD4 T cells, CD8 T cells, and macrophages. Velmeshev *et al*. [19] stated that in Autism Spectrum Disorder patients, cell types with common developmental lineages showed similar transcriptional changes. Grubman *et al*. [20] reported that Genome-Wide Association Study genes showed concordant up or down regulation in cell subclusters associated with Alzheimer’s disease, including microglia, astrocytes, and oligodendrocytes. Habermann *et al*. [21] identified that in multiple cell types, genes encoding for extracellular matrix components had been increased in idiopathic Pulmonary fibrosis (PF) lungs. The DE state correlation among cell types can potentially contribute scRNA-seq DE analysis to improve the performance; however, none of the existing methods have taken this correlation into account, resulting in low accuracy and power, especially for cell types with low abundance. In an earlier work, Chen *et al*. [22] reported the DE status correlation among cell types based on bulk data, and developed a method to incorporate such correlation in bulk data deconvolution problem.

In this article, we develop a novel statistical method to incorporate the cell type correlations into scRNA-seq DE analysis. The essence of the method is to model the gene expression through a Bayesian hierarchical model, where the correlations among cell type are characterized in the prior probabilities. The DE genes are then ranked and detected from the posterior probabilities. The main intuition of the method is that the inference for DE states for a gene in one cell type would be influenced by other cell types. Jointly modeling the expression in all cell types creates information sharing, which will improve the statistical inference. We name the proposed method “*s*ingle *c*ell *D*ifferential *E*xpression *TE*st with *C*ell *T*ype correlation (scDETECT).” In the following sections, we will first illustrate the DE correlation among cell types in real dataset, and then present the statistical model of the proposed method. Finally, we conduct comparative analyses between the proposed method and existing methods with simulated and real data to demonstrate the improved performance. Our proposed method is implemented in the latest version of scDETECT package, which is freely available at https://github.com/wwzhang-study/scDETECT.

## Materials and methods

### Data model for gene expression

We formulate the data model for gene expression under a linear model framework. Suppose the scRNA-seq data are generated for *N* subjects, each is characterized by several subject-specific covariates. Among the covariates, we want to test DE for some of them. Assume that the data include the measurement of the expression level of *G* genes in each cell, and all cells can be categorized into *K* types. The expression level of *g*th gene in *c*th cell of *k*th cell type for *i*th subject is denoted as ${X}_{gikc}$. For each cell type, we first aggregate the expression levels for individual cells to create ‘pseudo-bulk’ expression. Then for each cell type, we obtain a *G × N* pseudo-bulk expression matrix. We normalize the expression by library size and then take a log, using the lognormalize function in Seurat [23]. Let ${Y}_{gik}$ represent the normalized pseudo-bulk expression of *g*th gene in *i*th individual for *k*th cell type. We define ${Z}_{gk}$, a binary random variable representing the DE state of *g*th gene in *k*th cell type. ${Z}_{gk}=1$ means the *g*th gene in *k*th cell type is DE and ${Z}_{gk}=0$ otherwise. Given the DE states, we assume that the expression ${Y}_{gik}$ follows a normal distribution: ${Y}_{gik}\mid{Z}_{gk}\sim N\big({m}_{gik},{\sigma}_{gk}^2\big)$. We model the means by a linear model ${m}_{gik}={\mu}_{gk}+{C}_i^T{\beta}_{gk}+{Z}_{gk}{A}_i^T{\delta}_{gk}$. Here ${\mu}_{gk}$ represents the baseline expression level of gene *g* in cell type *k*; ${A}_i$ represent any interested factors to be tested such as gender, ethnicity, or disease status, which can be continuous or binary; ${C}_i={\left({C}_{i1},\dots, {C}_{iQ}\right)}^T$ represent other covariates of subject *i* that may also affect expression level beside the factor of interest. ${\beta}_{gk}$ and ${\delta}_{gk}$ are coefficients of ${C}_i$ and ${A}_i$, respectively.

The goal of our method is to compute the posterior probability $P\left({Z}_{gk}=1|{Y}_g\right)$ to determine DE state of each gene, and then identify DE genes accordingly. For that, we need to estimate ${\hat{\beta}}_{gk}$ and ${\hat{\delta}}_{gk}$ and ${\hat{\sigma}}_{gk}^2$ in order to obtain the observed data likelihood $P\left({Y}_g|{Z}_g\right)$. Then we can derive the posterior probability with Bayesian model.

Supplementary Fig. S1 shows the workflow of scDETECT, which consists of four main steps. Given the scRNA-seq data, the first step is to create a ‘pseudo-bulk’ gene count matrix for each cell type by aggregating the gene counts for cells belonging to a certain cell type. The second step is to construct a cell type hierarchical tree to represent the cell type correlations. The third step is to incorporate the cell type correlations in the calculation of the prior probabilities for DE. The fourth step is to compute the posterior probabilities based on the Bayesian model for determining the DE state of each gene. The other three steps are described in detail below.

### Create the cell type hierarchy

The correlation among cell types is represented by a hierarchical tree, which can be obtained by hierarchical clustering. It is important to note that the correlations are computed based on the change of expression instead of the baseline expression. We first perform DE test for each cell type using DESeq2, and obtain the test statistics for all genes in each cell type, represented by ${tstat}_k$ for cell type *k*. To reduce noises from non-DE genes, we filtered out some genes and only keep the ones satisfying $\left\{g:\mathrm{for}\ 1\le g\le G,\exists k\in \left\{1,\dots, K\right\},\left|{tstat}_{gk}\right|> threshold\right\}$. The default threshold used is 2.58 (corresponding to a *P*-value of .01) and can be defined by users in our software. Then the distance among cell types is defined with the following form:


$$ distance\left({k}_1,{k}_2\right)=\frac{1}{2}\left(1- cor\left({tstat}_{k_1},{tstat}_{k_2}\right)\right). $$

The stronger the correlation between cell types, the smaller the distance. The cell type hierarchical tree is then constructed based on this distance.

### The prior probabilities

For each gene *g*, we define a series of binary random variables representing DE states for all nodes of the hierarchical tree: ${Z}_g$ for leaf nodes and ${D}_g$ for non-leaf nodes. The joint probability of ${Z}_g$ and ${D}_g$ represents the correlation of DE states among cell types. The DE states of leaf nodes in cell type *k* is represented by ${Z}_{gk}$ with ${Z}_{gk}\sim Bernoulli\left({\pi}_k\right)$. The state of the *n*th nonleaf node at *l*th level of the tree is represented by ${D}_{g{\Phi}_{l,n}}$, where ${\Phi}_{l,n}$ denotes the set of cell types for all descendant leaf nodes. The root node is denoted as ${D}_{g{\Phi}_{0,1}}$. And ${D}_{g{\Phi}_{l,n}}\sim Bernoulli\left({\pi}_{\Phi_{l,n}}\right)$. In the entire hierarchical tree, we assume that any node must be state 0 when its parent node is state 0, while its state follows a Bernoulli distribution if the parent node is state 1. Thus, the conditional distribution of ${Z}_{gk}$ satisfies ${Z}_{gk}\mid{D}_{g{\Phi}_{l,n}}\sim Bernoulli\left({p}_k{D}_{g{\Phi}_{l,n}}\right)$, where ${D}_{g{\Phi}_{l,n}}$ is the parent node of ${Z}_{gk}$. Similarly, the conditional distribution of any nonleaf node satisfies ${D}_{g{\Phi}_{l,n}}\mid{D}_{g{\Phi}_{l^{\prime },{n}^{\prime }}}\sim Bernoulli\left({p}_{\Phi_{l,n}},{D}_{g{\Phi}_{l^{\prime },{n}^{\prime }}}\right)$, where ${D}_{g{\Phi}_{l^{\prime },{n}^{\prime }}}$ is the parent node of ${D}_{g{\Phi}_{l,n}}$. Finally, we assume that sibling nodes are mutually independent if their parent node is state 1.

The estimation of prior probability to be DE for any node is based on the *P*-values derived from DESeq2. Prior probabilities of any leaf node ${Z}_{gk}$ can be estimated as the proportion of genes deemed significant by DESeq2 in cell type *k* as following:


$$ {\hat{\pi}}_k=\frac{\sum_{g=1}^GI\left({pval}_{gk}< threshold\right)}{G}. $$

The prior probability for nonleaf nodes is estimated in a similar way, by just replacing leaf node with nonleaf node, which contains several cell types:


$$ {\hat{\pi}}_{\Phi_{l,n}}=\frac{\sum_{g=1}^GI\left(\underset{k\in{\Phi}_{l,n}}{\min}\left\{{pval}_{gk}\right\}< threshold\right)}{G}. $$

The conditional probability of leaf and nonleaf nodes are estimated by dividing prior probabilities by marginal probabilities of their parent nodes, which can be represented as following:


$${\hat{p}}_k=\frac{{\hat{\pi}}_k}{{\hat{\pi}}_{\Phi_{l,n}}},\ {\hat{p}}_{\Phi_{l,n}}=\frac{{\hat{\pi}}_{\Phi_{l,n}}}{{\hat{\pi}}_{\Phi_{l^{\prime },{n}^{\prime }}}}.$$

Then the joint probability of ${Z}_g=\left({Z}_{g1},\dots, {Z}_{gK}\right)$ and ${D}_g=\left({D}_{g{\Phi}_{0,1}},\dots, {D}_{g{\Phi}_{L,{n}_L}}\right)$ can be represented as the following form:


\begin{align*}\left({Z}_g,{D}_g\right)&=P\left({Z}_g|{D}_g\right)\times P\left({D}_g\right) =\Big\{{\prod}_{k=1}^KP\Big({Z}_{gk}| parent\left({Z}_{gk}\right)\Big\}\\&\quad\times \left\{{\prod}_{l=1}^L{\prod}_{n=1}^{n_l}P\left({D}_{g{\Phi}_{l,n}}| parent\left({D}_{g{\Phi}_{l,n}}\right)\right)\right\}\times P\left({D}_{g{\Phi}_{0,1}}\right)\\ &=\left({\prod}_{k=1}^K\left\{{\left[{\hat{p}}_k parent\left({Z}_{gk}\right)\right]}^{Z_{gk}}\bullet{\left[1-{\hat{p}}_k parent\left({Z}_{gk}\right)\right]}^{1-{Z}_{gk}}\right\}\right)\\&\quad\times\left({\prod}_{l=1}^L{\prod}_{n=1}^{n_l}\Big\{{\left[{\hat{p}}_{\Phi_{l,n}} parent\left({D}_{g{\Phi}_{l,n}}\right)\right]}^{D_{g{\Phi}_{l,n}}}\cdot \right.\\ &{\left[1-{\hat{p}}_{\Phi_{l,n}} parent\left({D}_{g{\Phi}_{l,n}}\right)\right]}^{1-{D}_{g{\Phi}_{l,n}}}\Big\}\bigg)\\&\quad\times\left({{\hat{\pi}}_{\Phi_{0,1}}}^{D_{g{\Phi}_{0,1}}}{\left(1-{\hat{\pi}}_{\Phi_{0,1}}\right)}^{1-{D}_{g{\Phi}_{0,1}}}\right).\end{align*}

### Data likelihood and posterior probability

For each cell type *k*, we fit two linear models when ${Z}_{gk}=1$ or ${Z}_{gk}=0$, where the model parameters including ${Z}_{gk}$, ${\hat{\mu}}_{gk}$, ${\hat{\beta}}_{gk}$, ${\hat{\delta}}_{gk}$, and ${\hat{\sigma}}_{gk}^2$ are estimated by ordinary least squares and maximum-likelihood estimation. Then we can obtain the observed data likelihood as:


\begin{align*} P\left({Y}_g|{Z}_g\right)&=\prod_{i=1}^N\prod_{k=1}^KP\left({Y}_{gik}|{Z}_{gk}\right)\\& =\prod_{i=1}^N\prod_{k=1}^K\left[\frac{1}{\sqrt{2\pi }{\hat{\sigma}}_{gk}}{e}^{-\frac{{\left({Y}_{gik}-{\hat{\mu}}_{gk}-{\boldsymbol{C}}_{\boldsymbol{i}}^{\boldsymbol{T}}{\hat{\boldsymbol{\beta}}}_{\boldsymbol{gk}}-{Z}_{gk}{\boldsymbol{A}}_{\boldsymbol{i}}^{\boldsymbol{T}}{\hat{\boldsymbol{\delta}}}_{\boldsymbol{gk}}\right)}^2}{2{\hat{\sigma}}_{gk}^2}}\right]. \end{align*}

Then the joint likelihood of ${Y}_g$, ${Z}_g$, ${D}_g$ can be derived as:


\begin{align*}P\left({Y}_g,{Z}_g,{D}_g\right)&=P\left({Y}_g|{Z}_g\right)\times P\left({Z}_g,{D}_g\right) \\ &=\prod_{i=1}^N\prod_{k=1}^KP\left({Y}_{gik}|{Z}_{gk}\right)\times P\left({Z}_g,{D}_g\right). \end{align*}

Summing over all combinations of $ {Z}_g $ and $ {D}_g $, we can get the marginal likelihood of $ {Y}_g $ as:


$$ P\left({Y}_g\right)=\sum_{\left({Z}_g,{D}_g\right)}P\left({Y}_g,{Z}_g,{D}_g\right). $$

Note that in the summation, there are ${2}^K$ different values for ${Z}_g$, and ${D}_g$ is fully determined given ${Z}_g$. Thus, there are ${2}^K$ terms in the summation.

Given ${Z}_{gk}=1$, we sum over the possible combinations of ${Z}_g$ and ${D}_g$ and obtain:


$$ P\left({Y}_g,{Z}_{gk}=1\right)=\sum_{\left({Z}_g,{D}_g\right)}P\left({Y}_g,{Z}_g,{D}_g\right)\times I\left({Z}_{gk}=1\right). $$

With these results, we can obtain the posterior probability of ${Z}_{gk}=1$ conditional on ${Y}_g$ based on Bayesian rule, as shown below


$$ P\left({Z}_{gk}=1|{Y}_g\right)=\frac{P\left({Y}_g,{Z}_{gk}=1\right)}{P\left({Y}_g\right)}. $$

### Simulation setting

We create data simulation based on real data, so that the simulated data resemble the real data characteristics. We obtain a dataset from Gene Expression Omnibus (GEO) with accession number GSE158055, which contains individuals of whole blood cells from healthy individuals and patients with various degrees of COVID-19 (Coronavirus Disease 2019). This dataset (referred to as the COVID-19 data hereafter) is used as a reference for the simulation. In the simulation, we generate 13,653 genes and a total of 200,000 cells from six cell types, and 20 subjects per group. The proportion of cells allocated to each cell type and sample is the same as the proportion of cells in the reference dataset. The true DE states of genes are simulated according to a hierarchical tree structure (Fig. 2a) with 20% DE genes in each cell type. We generate the DE state as ${D}_{g{\varPhi}_{0,1}}\sim Bernoulli\left({\pi}_{\varPhi_{0,1}}\right)$ for root node. Among genes with DE state 1, any of its child nodes is generated from ${D}_{g{\varPhi}_{l,n}}\sim Bernoulli\left({p}_{\varPhi_{l,n}}\right)$. The prior probability of DE state for each node is specified as the following: ${\pi}_{\left\{1,2,3,4,5,6\right\}}=0.5$, ${p}_{\left\{1,2,3,4\right\}}=0.667$, ${p}_{\left\{2,3,4\right\}}=0.8$, ${p}_1=0.6$, ${p}_{\left\{3,4\right\}}=0.833$, ${p}_2=0.75$, ${p}_3={p}_4=0.9$, ${p}_{\left\{5,6\right\}}=0.444$, ${p}_5={p}_6=0.9$. These data are chosen to mimic the real data.

Next, we simulate gene expression of cell type *k* in subject *i* using an NB model:


$$ {X}_{gik c}^{(j)}\sim NB\left({s}_{gik c}{\mu}_{gik}^{(j)},{\phi}_{gik}^{(j)}\right),c=1,2,\dots, {n}_{ik}, $$

$$ {\mathit{\log}}_2{\mu}_{gik}^{(j)}=\left\{\!\!\begin{array}{l}{\mathit{\log}}_2{m}_{gik},j=1\\{}{\mathit{\log}}_2{m}_{gik}+{lfc}_{gk},j=2\end{array}\right.\ . $$

Here *j* refers to the outcome group. ${s}_{gikc}$ is a size factor from the sequencing depth, which is simulated from a uniform distribution U(0, 0.12), resembling the real data. The baseline mean ${m}_{gik}$ is estimated from normalized expression values of reference dataset. The log2 fold change *lfc* is drawn from *N*(0.065,0.12^2^) in DE genes and from *N*(0, 0.001^2^) in non-DE genes. The dispersion ${\phi}_{gik}$ is simulated as a function of ${\mu}_{gik}:\frac{0.01}{\mu_{gik}}+0.05$. All parameter settings reflect the characteristics of the real data. Besides these specified parameter values, we also tried a range of other parameter values to test the robustness of the proposed methods. Details are provided in the Results section.

## Results

### Correlations of differential expression states among cell types from COVID-19 data

We explore the correlation of DE states among cell types in the COVID-19 data. COVID-19 has a total of eight cell types, CD4 (CD4 T-cell), CD8 (CD8 T-cell), B (B-cell), Mono (Monocyte), NK (Natural killer), DC (Dendritic cell), Plasma (Plasma-cell), and Mega (Megakaryocyte). We first apply DESeq2 to call DE between normal individuals and severe patients for each cell type. Then we compute the Pearson correlation coefficient of the test statistics from the DESeq2 tests to evaluate the pairwise correlation of DE states among cell types.

**Figure 1:**
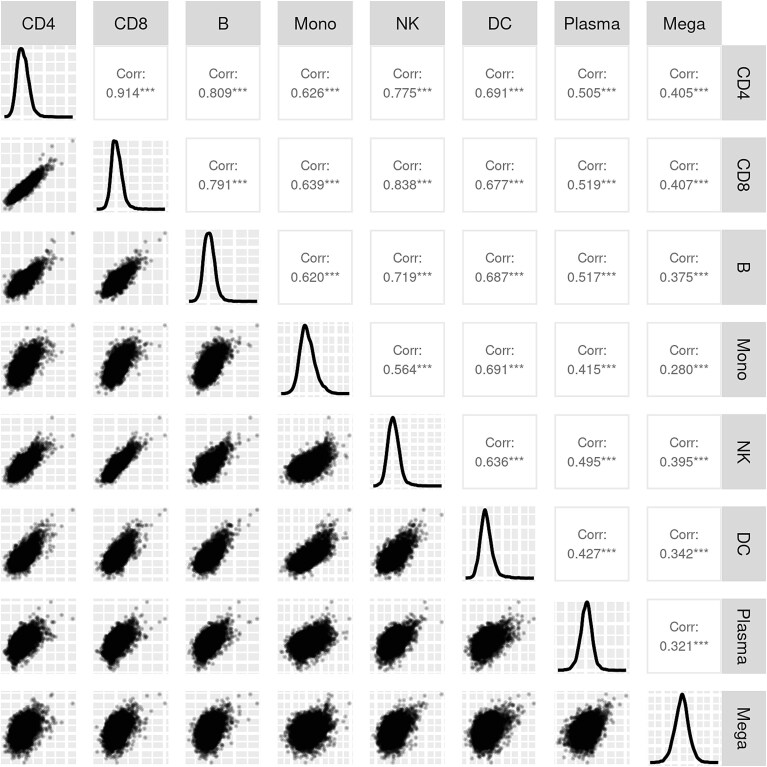
Correlation of DE status between cell types. X- and Y-axes represent test statistics from DESeq2 in corresponding cell types. Each point represents one gene. The diagonal part represents the probability density curve of the test statistics for each cell type. The Pearson correlation coefficients of test statistics are calculated and tested for significance. *** denotes *P*-value<.01.

The pairwise scatterplot of the correlation between cell types is shown in Fig. 1. As shown in the figure, the Pearson correlation coefficients for most cell types are over 0.6. This demonstrates that there is indeed strong correlations in DE states among cell types. In addition, the correlation strength between different cell types varies. For example, CD4, CD8, and B cells have stronger correlations with each other than others, with the coefficients over 0.8. In all, these results indicate a hierarchical correlation structure among all cell types.

### Simulation

#### scDETECT improves accuracy in differential expression gene detection

We first evaluate the DE calling performance of the proposed method. As a benchmark, we compare with several existing scRNA-seq DE methods including DESeq2, two-sample t-test, and Seurat-MAST. Among them, DESeq2 and t-test are conducted on pseudo-bulks, while Seurat-MAST is conducted on the single-cell level. The simulation settings are described before. Briefly, the simulation is based on a COVID-19 whole blood dataset (GEO accession number GSE158055) with six cell types: NK, B, CD4, CD8, Mono, and DC. We simulate expression levels of 13,653 genes across 20 samples per group in 6 cell types, with the parameters estimated from the real data to make sure the characteristics of simulated data similar to the real data.

**Figure 2:**
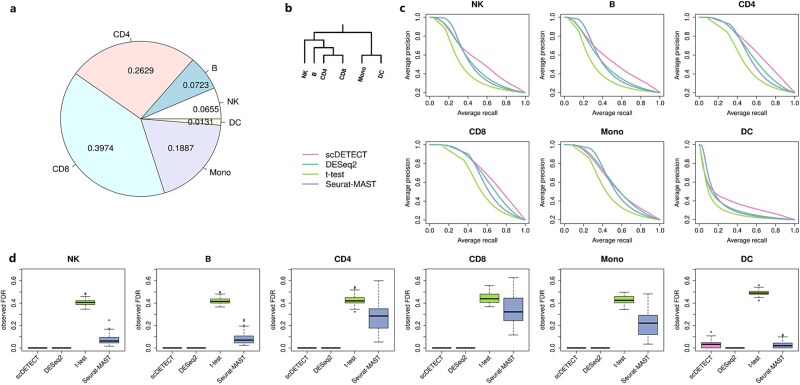
Simulation results for different methods of DE gene analysis. The single-cell expression data are generated in 6 cell types, with 20 samples in each group. (a) Proportion for each cell type. (b) Cell type hierarchy for the simulation. (c) PR curves for DE gene detection in six cell types from four methods (scDETECT, DESeq2, t-test, and Seurat-MAST). The results are averaged from 50 simulations. (d) Boxplots of FDR for DE gene detection with four methods. The genes are identified as DE when estimated FDR < 0.05 (DESeq2, t-test, and Seurat-MAST); posterior probability of DE > 0.95 (scDETECT). The boxplots show the FDRs from 50 simulations.

In each cell type, we set 20% of all genes to be DE genes. Then we set different levels DE gene correlation among the six cell types using a predefined hierarchical tree structure for simulation (Fig. 2b). The strongest correlation between CD4 and CD8 as well as Mono and DC is simulated with 90% overlapped DE genes. B is simulated to have a weaker correlation with CD4 and CD8 with 75% overlapped DE genes, and NK correlates with them with 60% DE genes overlapped. The weakest correlation is simulated between NK, B, CD4, CD8 and Mono, DC. There are 40% overlapped DE genes between any two of them. Then we apply the four methods DESeq2, t-test, Seurat-MAST, and scDETECT to detect DE genes.

Firstly, we compare the four methods in their precision-recall (PR) curves (Fig.  2c). The results summarized over 50 simulations show that compared with other three methods, scDETECT has improved accuracy of DE genes detection in almost all cell types. However, the extent of improvement varies among cell types. The improvement of cell types with smaller proportions (NK, B, and DC) is greater than those from cell types with larger proportions (CD4, CD8, and Mono). The primary reason is that NK and B are clustered with CD4 and CD8 (with large proportion) with strong correlation (0.6 and 0.75), so that they can borrow information from cell types with large proportion. In contrast, the improvement of Mono is smaller where the PR curve is nearly identical with that from DESeq2. That is because Mono is clustered with DC, the cell type with the smallest proportion (0.01) with strong correlation (0.9) but has weak correlation (0.4) with CD4 and CD8, making it hard to borrow information. Table 1 presents the AUC values (the area under PR curves) of the four methods on six cell types, which are consistent with the results in Fig.  2c. The results in Table 1 are the average of 50 simulations.

**Table 1:** AUC values of DE gene detection for each cell type under four methods

Cell types	scDETECT	DESeq2	t-test	Seurat-MAST
NK	0.563	0.533	0.460	0.533
B	0.564	0.510	0.441	0.517
CD4	0.696	0.647	0.559	0.627
CD8	0.729	0.690	0.601	0.666
Mono	0.608	0.613	0.534	0.607
DC	0.381	0.333	0.304	0.343

Then we compare the false discovery rate (FDR) control of four methods from 50 simulations (Fig.  2d). We set estimated FDR of 0.05 as a cutoff for DESeq2, t-test, and Seurat-MAST, and posterior probability of DE 0.95 as a cutoff for scDETECT. The results show that scDETECT and DESeq2 control FDR well. In fact, they are on the more conservative side since the FDRs are always kept nearly 0. By contrast, t-test always has the highest FDR, which keeps >0.4 in each cell type. This is because t-test is performed at cell level and the large number of cells artificially increases the statistical power. That is an undesirable effect, since it makes many non-DE genes with very small difference between groups to be detected as DE genes, which provides too many false positives. The FDR for Seurat-MAST is also high in some cell types such as CD4, CD8, and Mono, but is better than t-test overall due to better modeling and test procedure. Overall, the simulation results demonstrate that scDETECT provides better DE test accuracy while controls the type I error well.

### Robustness against mis-specified cell type hierarchy

We will encounter different correlation patterns of DE states under different conditions in practical application. Some cell types show strong correlation with each other in one situation but not in another. Next, we compare the four methods under different cell type hierarchical structures in order to further evaluate the performance and robustness of scDETECT. We simulate data for the six cell types as before and set four different scenarios of cell type correlation relationships (Supplementary Fig. S2a–d).

The simulation results show that scDETECT does not have any advantage when all the cell types are independent with each other (Supplementary Fig. S2a). This makes sense since when cell types are independent, there is not information sharing so that modeling the correlation does not help. When cell types are correlated strongly, scDETECT has great improvement over other methods (Supplementary Fig. S2b–d). However, the improvements in CD4 under the second scenario (Supplementary Fig. S2b) and CD8 under the third scenario (Supplementary Fig. S2c) are not as significant as others. Because the cell types with large proportion are only correlated with the ones with smaller proportions, while independent with others. Then it cannot borrow much useful information.

We further compare scDETECT using mis-specified tree structures with that using true tree structure, which evaluates the robustness of scDETECT. The results show that scDETECT is robust to mis-specified tree structures in general. The performance variation often appears when any cell type is mis-clustered with those with smaller proportions. More detailed discussions are provided in Supplementary data 1.

Finally, We compared the efficiency of scDETECT with MAST, which is specifically designed for scRNA-seq DE analysis. For a simulation with 40 samples, scDETECT takes about 64.89 s, and Seurat-MAST takes 136.54 s for one run on a Intel Core with i7 1.8 GHz CPU and 8 GB RAM. scDETECT provides superior computational performance than Seurat-MAST.

### Real data analysis

For real data, we also use dataset GSE158055. Different from simulated data analysis, here we extracted healthy individuals and severe COVID-19 patients from the whole dataset. After filtering out samples free of other diseases, those from frozen blood, and those from the 10× Genomics sequencing platform, we got five normal samples and eight disease samples. We apply scDETECT, DESeq2, t-test, and Seurat-MAST to call DE between normal versus disease in eight cell types: NK, B, CD4, CD8, monocytes, DC, Mega, and Plasma cells. In each cell type, we filtered out genes expressed in <10 cells.

#### Overlap of differential expression genes obtained by different methods

**Figure 3:**
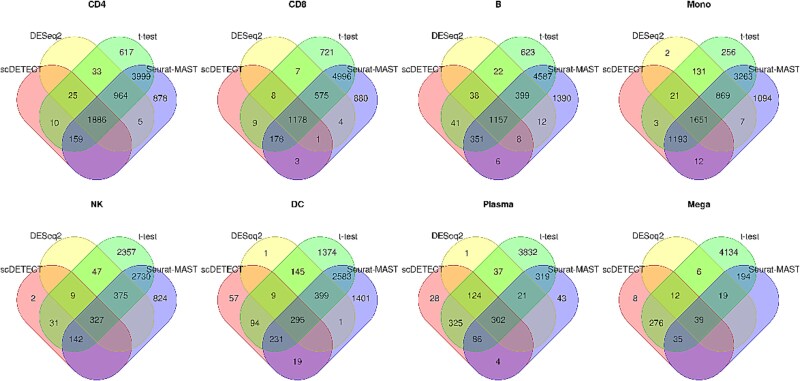
Venn diagram showing overlap of DE genes identified by four methods in eight cell types.

We first assess the overlap of DE genes detected by all four methods in eight cell types by a Venn diagram (Fig.  3). Results show that scDETECT and DESeq2 have large overlaps, and there are few DE genes identified uniquely by scDETECT. In most cell types, 65% of the DE genes detected by scDETECT are recognized as DE by DESeq2, and even up to 87% in Plasma, with the lowest proportion being 35% in DC. On the other hand, t-test, and Seurat-MAST have many shared DE genes, but most of their DE genes are unique and not identified by scDETECT and DESeq2. This makes sense because scDETECT and DESeq2 are based on pseudo-bulk, but t-test, and Seurat-MAST are based on individual cells.

To further compare the ranking of the DE genes from different methods, we rank the posterior probabilities from scDETECT, the statistics from DESeq2, and the LFCs exported by Seurat-MAST in descending order and take out DE genes of top 500, respectively. The Venn diagram is shown in Supplementary Fig. S3. We see that in many cell types, the proportion of DE genes detected by scDETECT among all DE genes from DESeq2 have decreased compared with the former result. However, the overlap rate between scDETECT and t-test, Seurat-MAST has increased, even exceeds that between scDETECT and DESeq2. And the proportions of DE genes uniquely identified by t-test have also decreased among DE genes of a higher level of significance. This indicates that top-ranked DE genes from t-test and Seurat-MAST are representative, though there are many false positives in lower ranked genes, especially from t-test.

#### Evaluate the type I error control in real data

We perform a permutation test in the real data to further evaluate the type I error control of different methods. We shuffle the clinical conditions of sample labels randomly to get the permutation of real data, and then apply different methods to call DE genes. The numbers of DE genes are shown in Supplementary Fig. S4. We find that very few genes are identified by scDETECT and DESeq2 from the permuted dataset. However, many DE genes are identified by t-test, which is more than half of that on the original data. For Seurat-MAST, there are also many DE genes in some cell types. We also compute the overlap of DE genes from permuted and original datasets. The boxplot of the overlap rates in all method are shown in Supplementary Fig. S5. We can see that the rates from both scDETECT and DESeq2 are very close to zero. The rates from t-test and Seurat-MAST are much higher, even >50% particularly from t-test. This suggests that many DE genes detected by t-test and Seurat-MAST on the original data are false discoveries. These results agree with the high FDR rates reported in previous section.

#### Impact of the sample sizes

**Figure 4:**
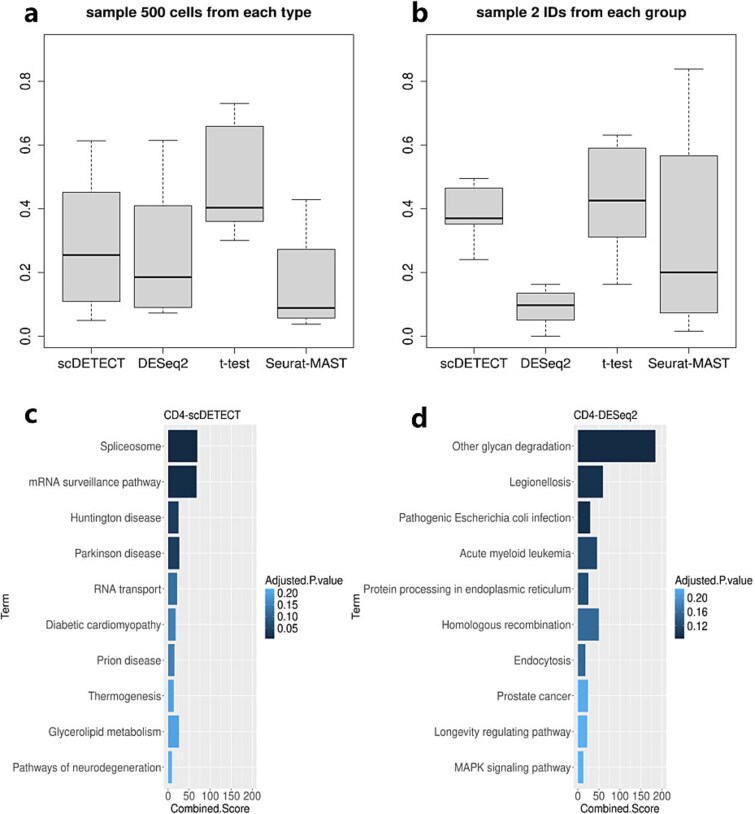
Boxplots of overlap rates between DE genes detected from subsampled datasets and the original dataset: (a) 500 cells sampled from each cell type. (b) Two individuals sampled from each group. Top 10 KEGG pathways enriched by DE genes: (c) detected uniquely by scDETECT but not by DESeq2, (d) detected uniquely by DESeq2 but not by scDETECT.

Next, we evaluate the robustness of the methods with different cell and sample numbers. In real-world applications, we wish the DE analysis results not overly affected by the sample size, including cell or sample numbers. The DE analysis method, i.e. robust against the sample size is more desirable. Here, we create two scenarios to evaluate the impacts of cell or sample numbers: randomly pick 500 cells from each cell type and randomly pick 2 samples from each group. We then call DE on these new datasets in the two scenarios and evaluate the overlaps of DE genes from the original data. High overlap means that the method is more robust. The overlap rates from such analyses are shown in Fig.  4a and b. We find that t-test has the highest rate in both cases. However, the result in Supplementary Fig. S5 indicates that DE result from t-test has very high false positives. Thus, its high overlap is achieved at the cost of high false positive rate and not reliable. In methods controlling false positive well (scDETECT and DESeq2 according to Supplementary Fig. S5), scDETECT has higher robustness. Comparing with Seurat-MAST, scDETECT also has advantage in robustness overall. These results demonstrate that scDETECT is less affected by the sample size, which is a desirable feature.

#### Enrichment analysis of differential expression genes

To evaluate the biological implication, we perform enrichment analyses to compare the DE genes detected by scDETECT and DESeq2. We obtain DE genes uniquely detected by scDETECT but not by DESeq2, and those uniquely detected by DESeq2 but not by scDETECT, then perform enrichment analyses using enrichR (Fig.  4c and d and Supplementary Figs. S6–S12) [24]. Here we present the results of the major four cell types: CD4, CD8, B, and Mono.

For only scDETECT-detected DE genes, the Spliceosome and mRNA surveillance pathway have been reported to be significantly enriched in CD4 and CD8 and many other cell types. As previous publication showed, NSP16, one kind of SARS-CoV-2 (Severe Acute Respiratory Syndrome Coronavirus 2) protein, acts to suppress global mRNA splicing in SARS-CoV-2-infected human cells thereby suppressing the immune defenses [25]. The virus even creates a cap structure to simulate the mRNA modification process of human thus escaping the surveillance of immune system [26]. Besides, there are many other Kyoto Encyclopedia of Genes and Genomes (KEGG) pathways reported having influence on COVID-19. Endocytosis is an important pathway for SARS-CoV-2 entering host cells [27]. T cell receptor is crucial in T cell-mediated virus recognition and clearance [28, 29]. The pathways of EGFR, which belong to the ErbB family, are activated in response to virus infection. Studies have shown that inhibition of EGFR signaling reduces infection of SARS-CoV-2 (Severe Acute Respiratory Syndrome Coronavirus 2) [30]. RNA transport pathways would be hijacked by SARS-CoV-2 and synthesize viral proteins in the cytoplasm, thus hindering host cell’s ability to mount an effective immune response [31]. And the ubiquitin-proteasome system would also be hijacked by SARS-CoV-2 to weaken host’s immunity [32, 33]. The renal cell carcinoma, neurotrophin, and HTLV-1 pathways affect the symptom of COVID-19 in aspect of regulation of pro-inflammatory cytokines [34–36], and the TCA and insulin pathways affect through host metabolic inhibition [37, 38].

For only DESeq2 detected DE genes, many significantly enriched pathways are related to other diseases like Parkinson disease, Alzheimer disease, and prion disease. This is because these diseases are similar to COVID-19 in immune regulation, which makes they have many common DE genes. The most significant pathway in Mono is the Oxidative phosphorylation pathway. Researchers have shown that the intermediates originated from glucose will be processed by oxidative phosphorylation to produce ATP, thus satisfying the energy needs for cells to combat virus infection [39].

To test the robustness of our method, we also downloaded the PBMC Lupus data from GEO repository under the accession ID GSE96583 to repeat the above analysis in C.1–C.3. The PBMC Lupus data contain 24 samples from 16 individuals. There are eight control samples from eight individuals with systemic lupus erythematosus (SLE) disease, eight control samples, and eight IFN-beta stimulated samples from another eight individuals with SLE disease. In each sample, there are seven cell types: B cells, CD14 + Monocytes, CD4 T cells, CD8 T cells, DCs, FCGR3A + Monocytes, and NK cells. We apply scDETECT, DESeq2, t-test, and Seurat-MAST to call DE between normal versus disease in the above seven cell types. We assess the overlap of DE genes detected by all four methods in seven cell types, the overlap of top 500 DE genes detected by all four methods in seven cell types, the DE gene numbers of the four methods for all cell types in permutation data, the overlap rates between DE genes detected from permutated datasets and the original dataset, the overlap rates between DE genes detected from subsampled datasets and the original dataset, respectively. Results are shown in Supplementary Figs. S13–S17. The results are consistent with those obtained using the COVID-19 dataset, indicating that scDETECT has good generalization ability and robustness.

#### Rediscovery rate by different methods

In order to evaluate the reproducibility of DE methods, we calculated the rediscovery rate (RDR) by [40]. The RDR is defined as *R_v_*/*R_t_. R_t_* is the number of DE genes in training sample, and *R_v_* as those which are retested in the validation sample. We take the COVID-19 dataset (GSE158055) as the training sample and PBMC Lupus data (GSE96583) as the validation sample. These two datasets share five common cell types: CD4 cells, CD8 cells, B cells, NK cells, and DCs. The results are shown in Supplementary Table S1. It is evident that, for all cell types, the RDR values by t-test are the highest among all methods, followed by scDETECT. But the RDR values by scDETECT are higher than those of DESeq2 and Seurat-MAST.

## Discussion

In this paper, we develop a novel statistical method called scDETECT for scRNA-seq differential expression that incorporates the DE state correlations among cell types. The idea of our work is motivated from the observation from real data, which show strong correlations of DE states among cell types. scDETECT is based on a Bayesian hierarchical model. It first constructs a cell type hierarchical tree to represent the cell type correlations, and then incorporates the correlation in the calculation of the prior probabilities for DE. After parameter estimation in data model, the posterior probabilities are then derived based on the Bayesian model for determining the DE state of each gene.

The comparative analysis on simulated data demonstrates that scDETECT improves the accuracy in DE gene detection, especially for cell types with small proportions. Besides, it controls the FDR well. When cell types have different correlation patterns, it shows various performance. We can see that scDETECT does not have any superiority over other methods when all cell types are independent. These results further demonstrate that the idea of using a hierarchical tree to get prior probabilities works well only on dataset with correlations on DE states among cell types. In addition, we find that the mis-specified cell type hierarchy has caused some deviation in the result of scDETECT. But overall, it is robust and still has improvements over other methods. In real data analysis, we discover that scDETECT possesses a high level of reliability and stability. For the enrichment analysis, it is shown that the biological implications of the pathways for scDETECT can better reflects the immune response mechanism of SARS-CoV-2 infection, which provides more practical guidance for effective treatments of COVID-19. In all, we demonstrate that accounting for cell type correlations improves the DE analysis in scRNA-seq.

scDETECT is a pure statistical tool with functionalities to better incorporate the biological context to improve data analysis results. From the biological perspective, we believe that the correlations among cell types mostly reflect biological relationships, and there have been numerous researches report the cofunctionalities among cell types [41–46]. There could be spurious correlations in the data that bares no biological meaning. However from the method point of view, as long as the correlation exists, it can facilitate data analysis.

In this work, we only show the results from a two-condition comparison in the real data analyses. However, our model is flexible enough to test the effect for continuous covariates. With the increasing application of scRNA-seq in large-scale population-level studies, our method provides a tool for better analysis for data from more complex experimental designs. In addition, the modeling of cell type correlations is also important in other single-cell assays, such as Single-cell Assay for Transposase-Accessible Chromatin using sequencing or spatial transcriptomics. That is a direction of our future research.

**Key Points**

- Differential expression (DE) is one of the most important analyses in single-cell RNA-seq (scRNA-seq). The existing methods perform DE analysis for each cell type separately after cell clustering and cell type annotation. However, due to similarity of cell types, the DE states often have strong correlation among different cell types. Existing methods ignore such correlation, resulting in low accuracy, and statistical power.
- In this paper, we develop a novel statistical method called ***s*ingle *c*ell *D*ifferential *E*xpression *TE*st with *C*ell *T*ype correlation** (scDETECT) for scRNA-seq DE that incorporates the DE state correlations among cell types. The essence of the method is to model the gene expression through a Bayesian hierarchical model, where the correlations among cell type are characterized in the prior probabilities. The DE genes are then ranked and detected from the posterior probabilities.
- Simulation and real data studies show that scDETECT significantly improves the accuracy and statistical power compared with existing methods.

## Supplementary Material

Supplementary_Materials_bbaf556

## Data Availability

The proposed method scDETECT is implemented as an R package at https://github.com/wwzhang-study/scDETECT.
