# Supplementary material for: scDETECT: a novel statistical model accounting for cell type correlation in single-cell RNA-seq differential expression analysis
Source: Brief Bioinform. 2025 Oct 27;26(5):bbaf556. doi: 10.1093/bib/bbaf556 (PMC12554637; doi:10.1093/bib/bbaf556)
Supplement: Supplementary_Materials_bbaf556 [file supplementary_materials_bbaf556.docx]

Supplementary Materials for “scDETECT: a novel statistical model accounting for cell type correlation in single cell RNA-seq differential expression analysis”

Yuhan Xu^1,2^, Weiwei Zhang^3*^, Hao Wu^1,4*^

^1^Faculty of Computer Science and Control Engineering, Shenzhen University of Advanced Technology, Shenzhen, Guangdong 518055, China,

^2^School of Statistics and Data Science, Nankai University, Tianjin 300171, China,

^3^School of Mathematics Information, Shaoxing University, Shaoxing, Zhejiang 312000, China,

^4^Institute of Advanced Computing and Digital Engineering, Shenzhen Institute of Advanced Technology, Chinese Academy of Sciences, Shenzhen, Guangdong 518055, China.

^*^To whom correspondence should be addressed.

**Supplementary Figure S1.** The flowchart of scDETECT algorithm.


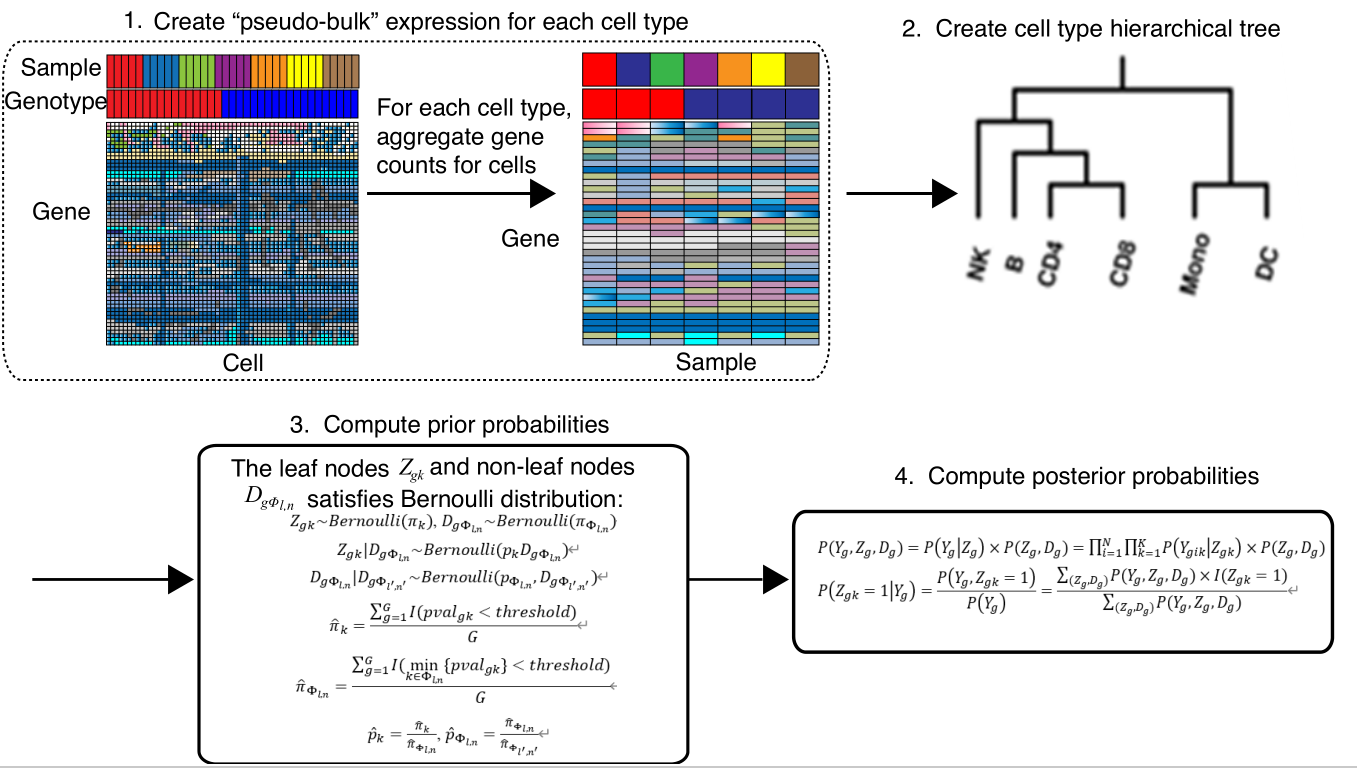


**Supplementary Figure S2.** PR curves under different correlation patterns of DE states. The simulation is conducted of DE gene analysis for six cell types (NK, B, CD4, CD8, Mono, DC) under four various DE patterns (**a.** all cell types are independent; **b.** only NK, B, CD4 are correlated, CD8, Mono, DC are independent; **c.** CD8, Mono, DC are correlated, NK, B, CD4 are independent; **d.** all cell types are correlated). The results are summarized from 50 simulations.

**

**

**Supplementary Figure S3.** Venn diagram showing overlap of top 500 DE genes detected by scDETECT, DESeq2, t-test and Seurat-MAST for all cell types. The order of DE genes are ranked by the posterior probabilities for scDETECT, the statistics for DESeq2 and t-test, and the LFCs for Seurat-MAST.

**
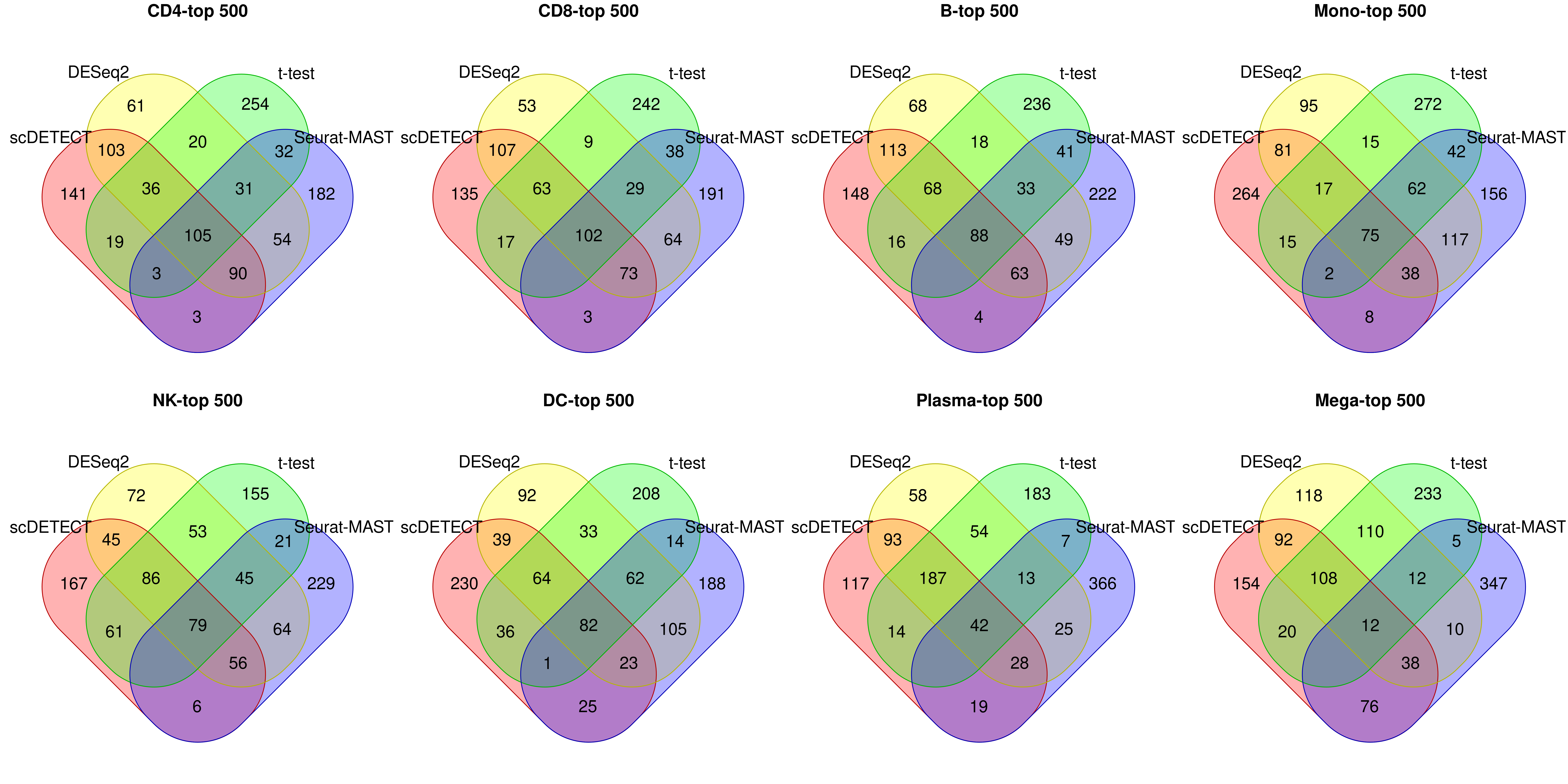
**

**Supplementary Figure S4.** Bar plots of the DE gene numbers of the four methods for all cell types in permutation data.


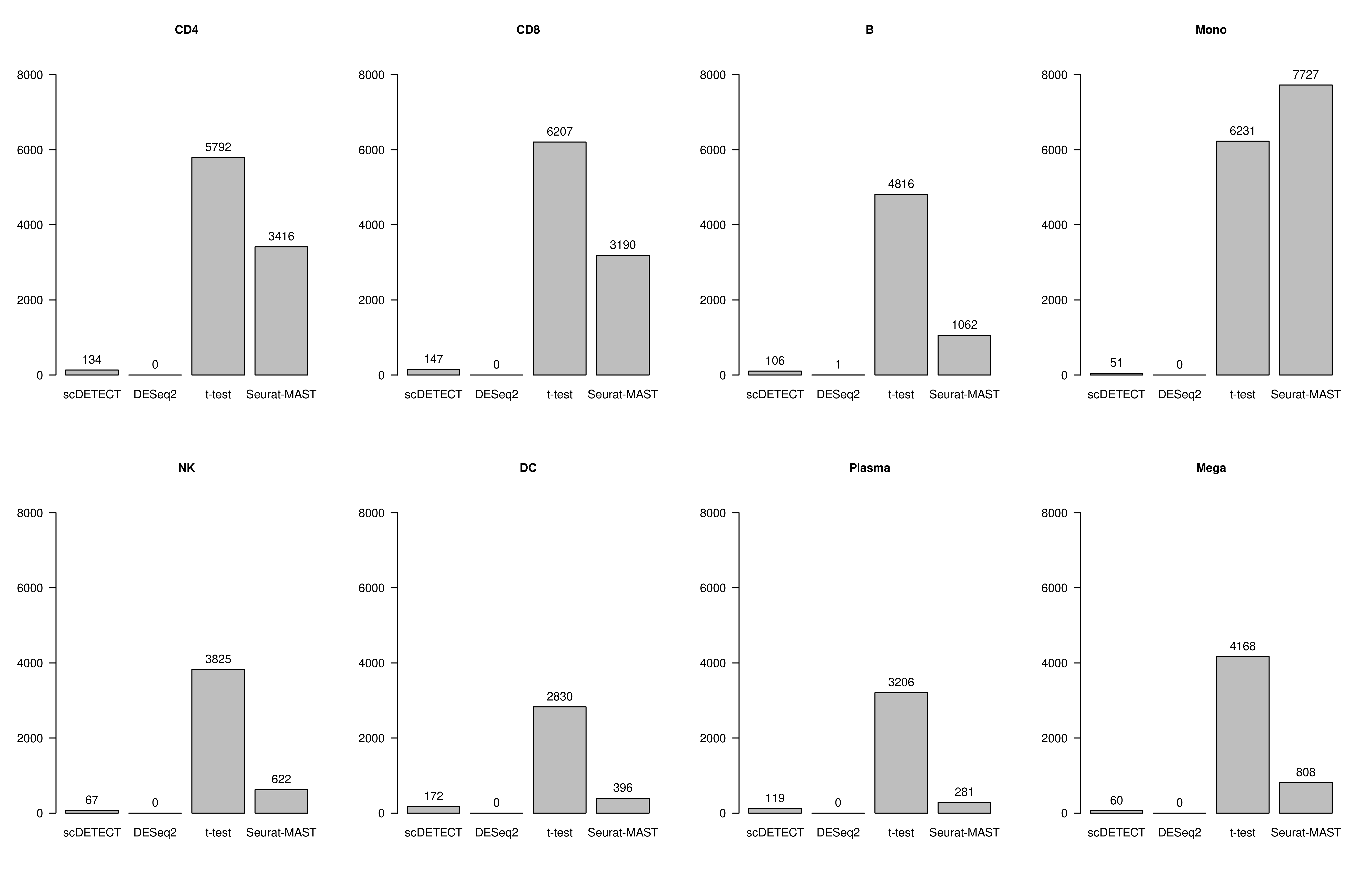


**Supplementary Figure S5.** Boxplot of overlap rates between DE genes detected from permutated datasets and the original dataset.

**
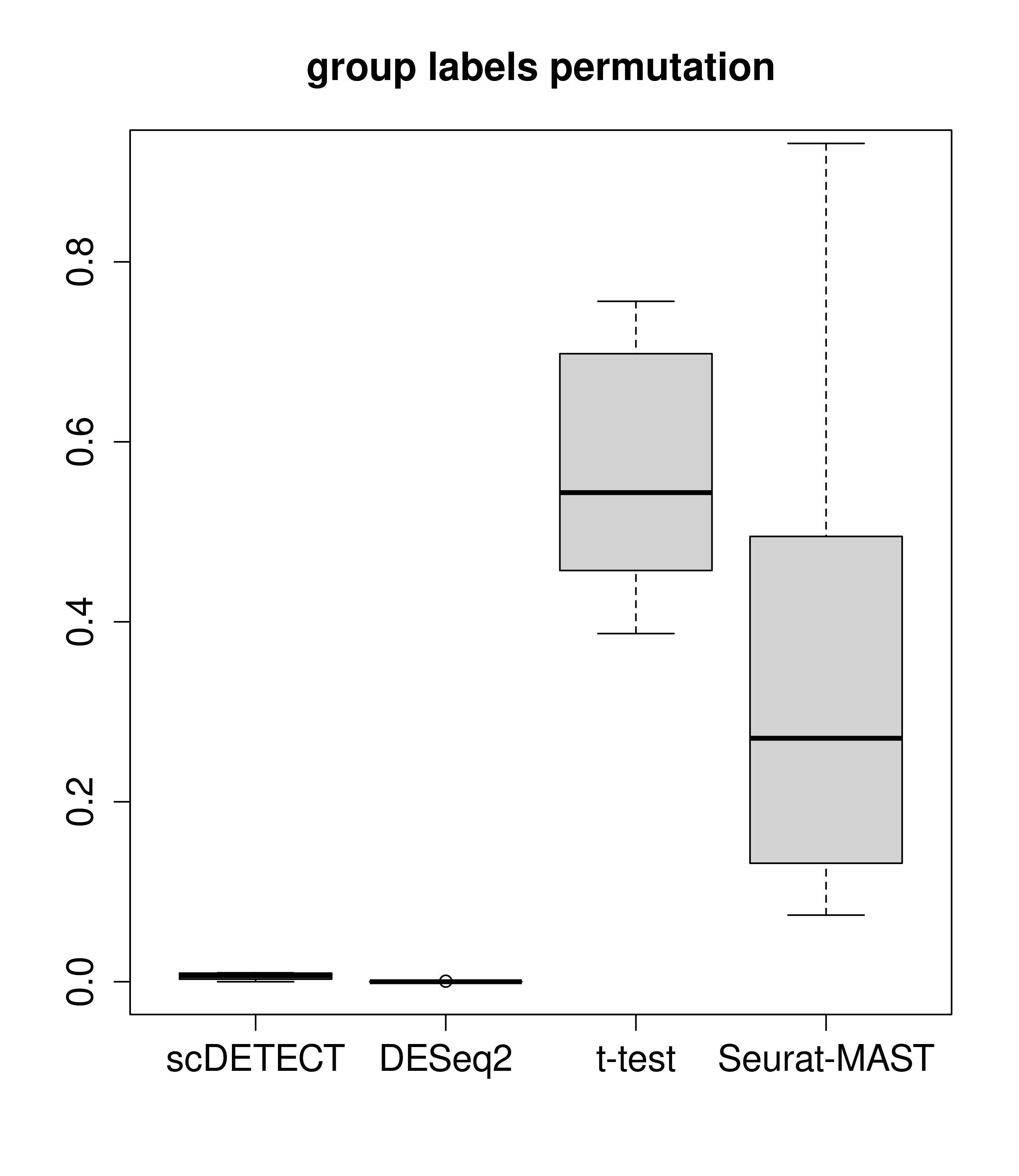
**

**Supplementary Figure S6. a.** KEGG pathways enriched by DE genes detected uniquely by scDETECT in CD8, but not by DESeq2. **b.** Top ten pathways enriched by DE genes detected uniquely by DESeq2 in CD8, but not by scDETECT.

**
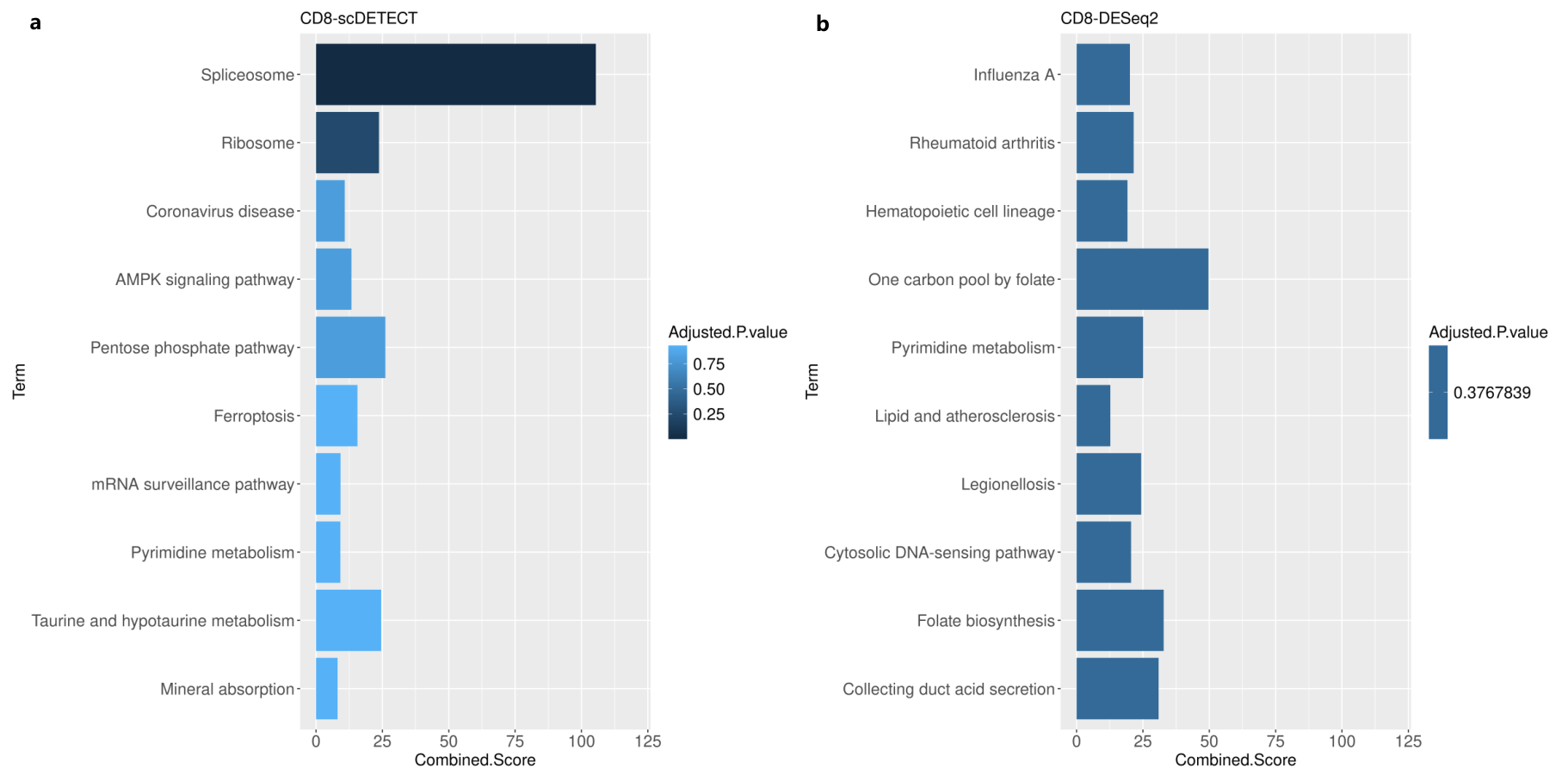
**

**Supplementary Figure S7. a.** KEGG pathways enriched by DE genes detected uniquely by scDETECT in B, but not by DESeq2. **b.** Pathways enriched by DE genes detected uniquely by DESeq2 in B, but not by scDETECT.

**
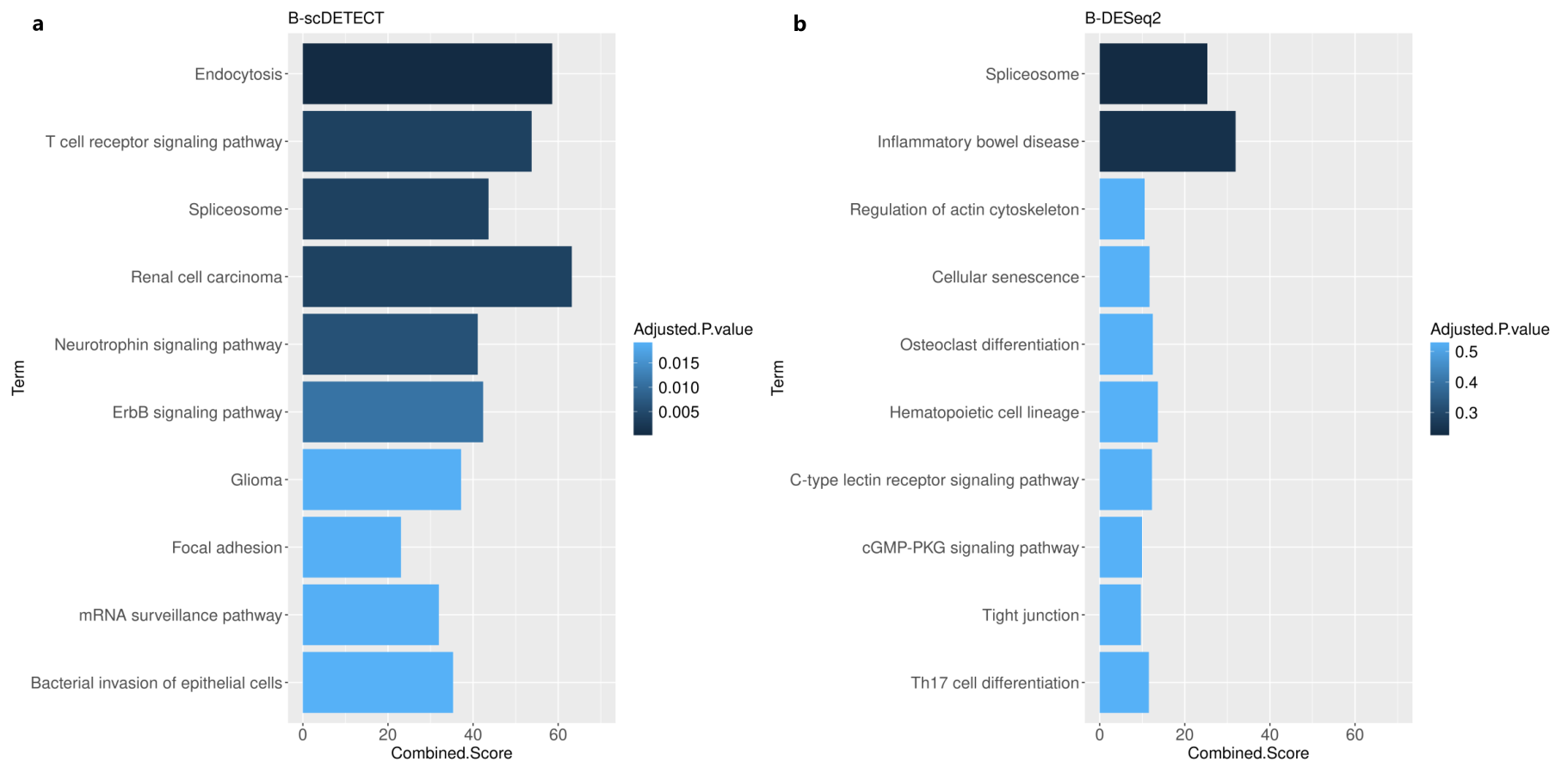
**

**Supplementary Figure S8. a.** KEGG pathways enriched by DE genes detected uniquely by scDETECT in Mono, but not by DESeq2. **b.** Pathways enriched by DE genes detected uniquely by DESeq2 in Mono, but not by scDETECT.

**
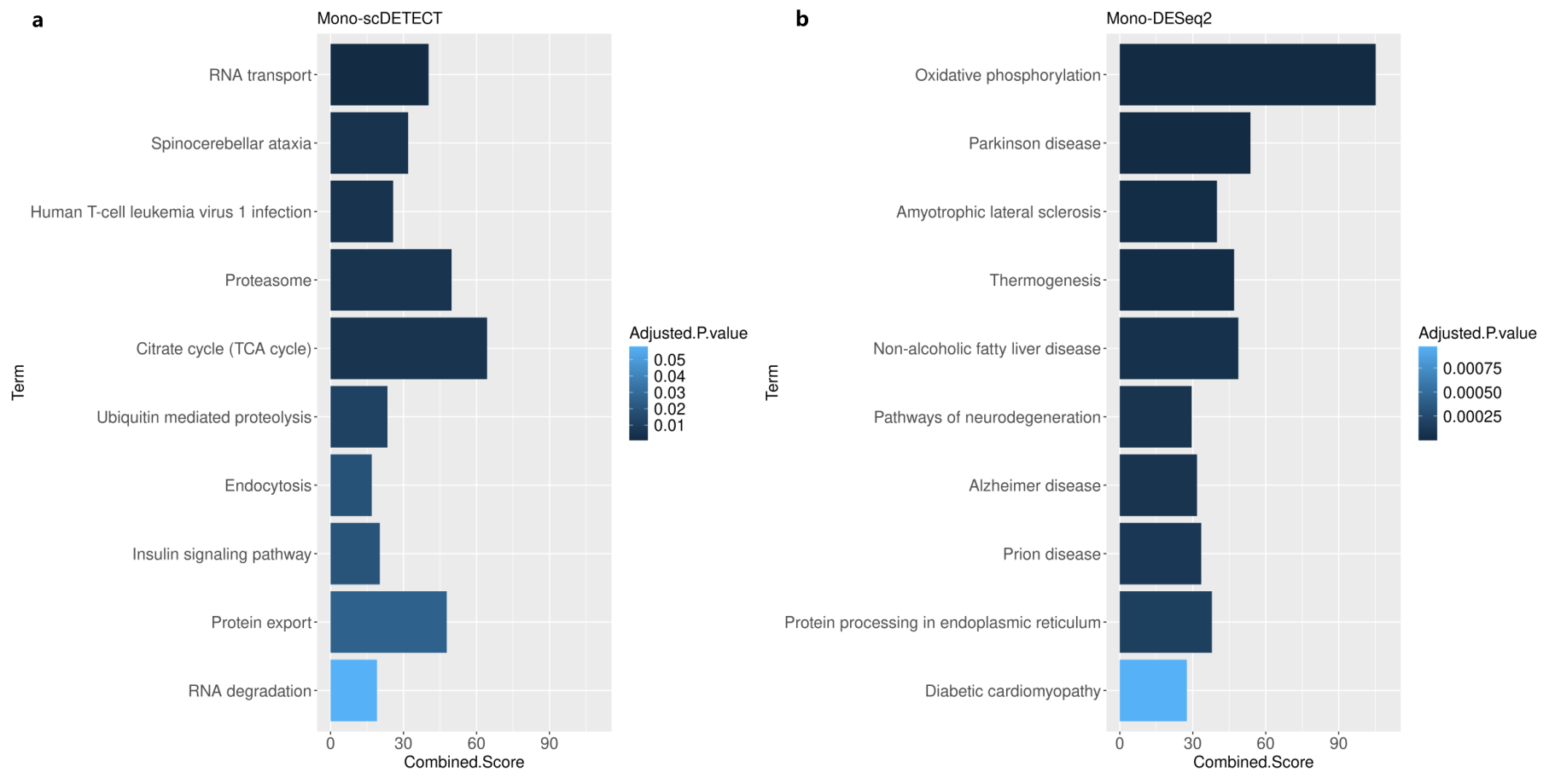
**

**Supplementary Figure S9.** **a.** KEGG pathways enriched by DE genes detected uniquely by scDETECT in NK, but not by DESeq2. **b.** Pathways enriched by DE genes detected uniquely by DESeq2 in NK, but not by scDETECT.

**
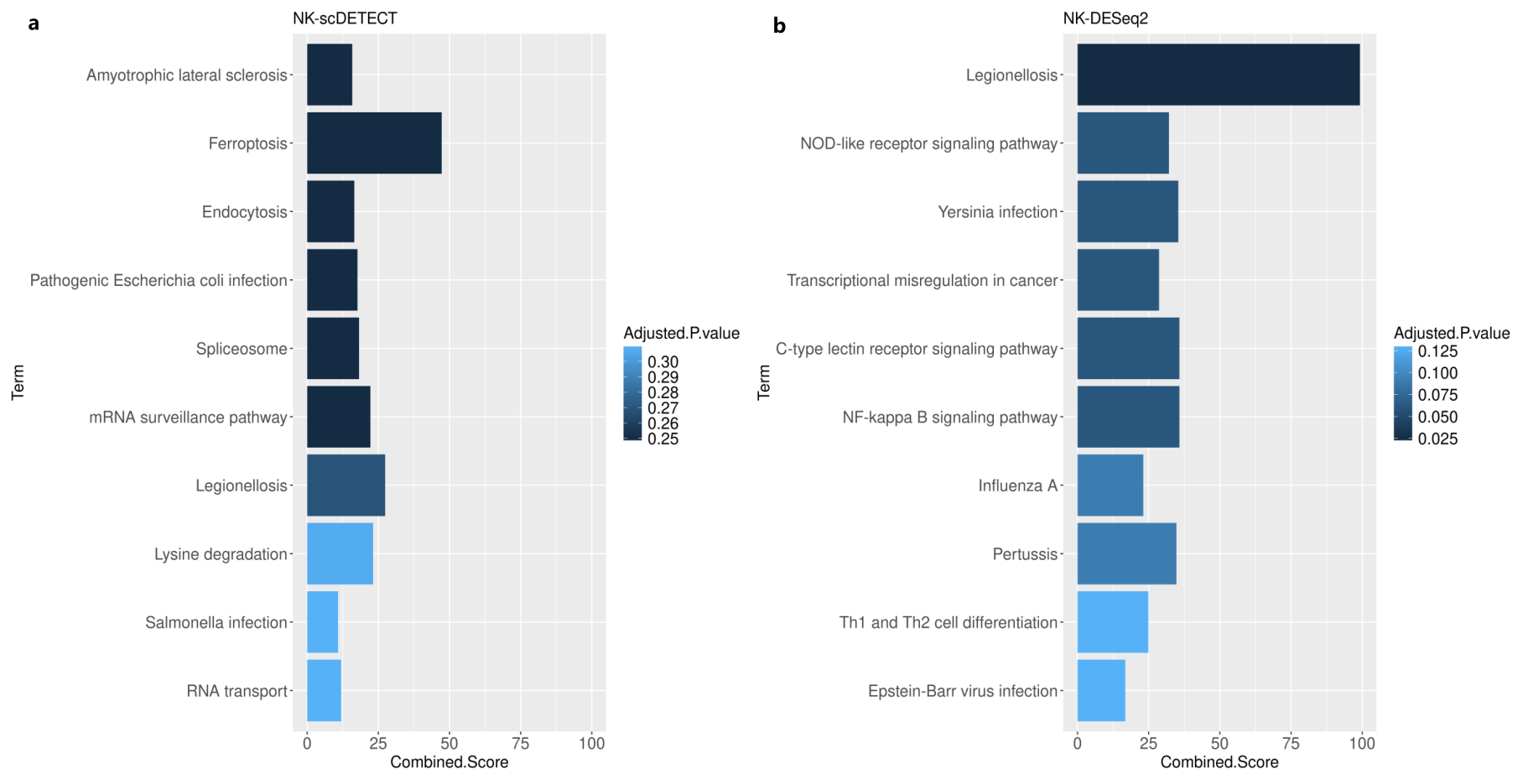
**

**Supplementary Figure S10.** **a.** KEGG pathways enriched by DE genes detected uniquely by scDETECT in DC, but not by DESeq2. **b.** Pathways enriched by DE genes detected uniquely by DESeq2 in DC, but not by scDETECT.

**
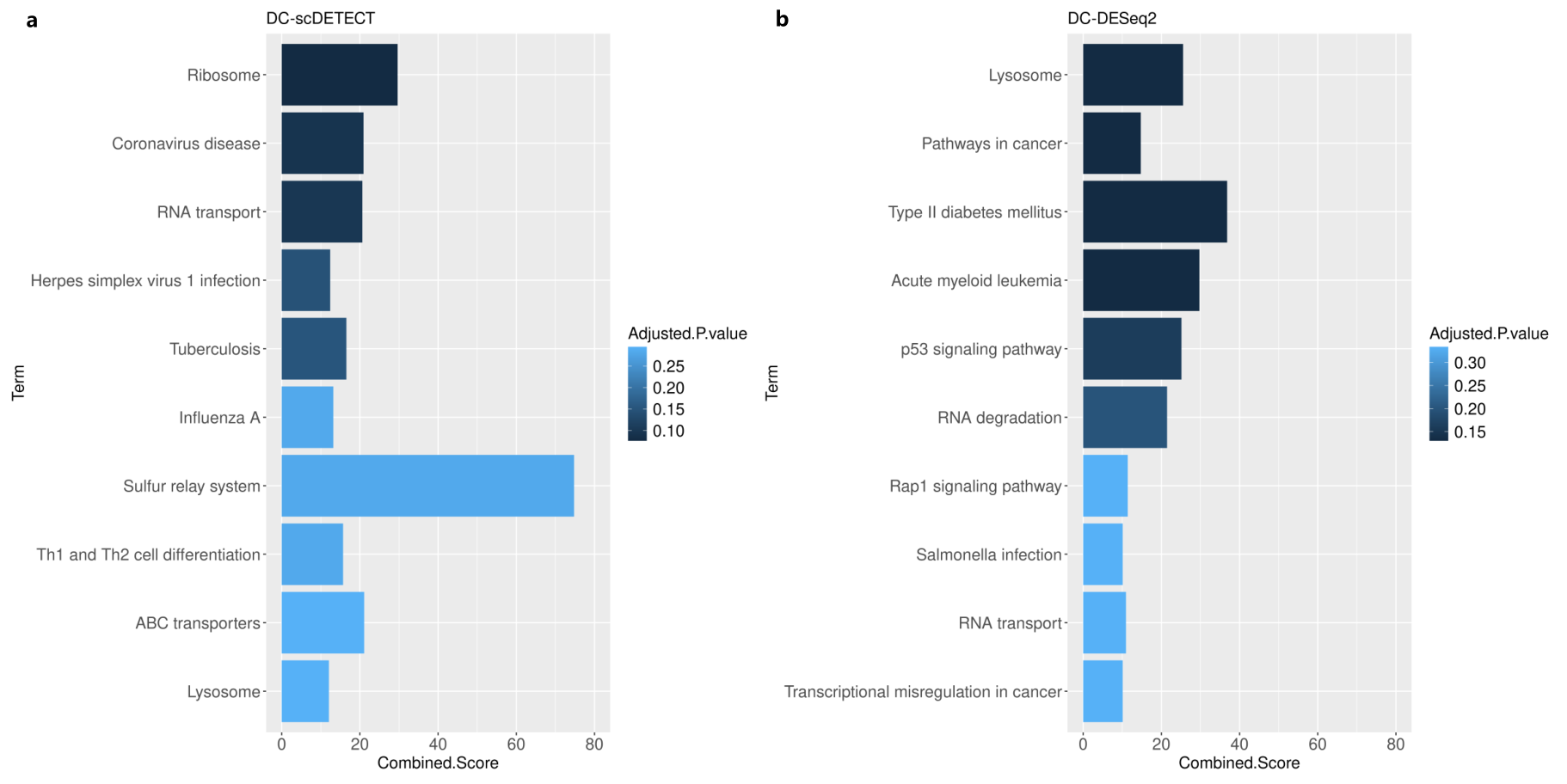
**

**Supplementary Figure S11. a.** KEGG pathways enriched by DE genes detected uniquely by scDETECT in Plasma, but not by DESeq2. **b.** Pathways enriched by DE genes detected uniquely by DESeq2 in Plasma, but not by scDETECT.

**
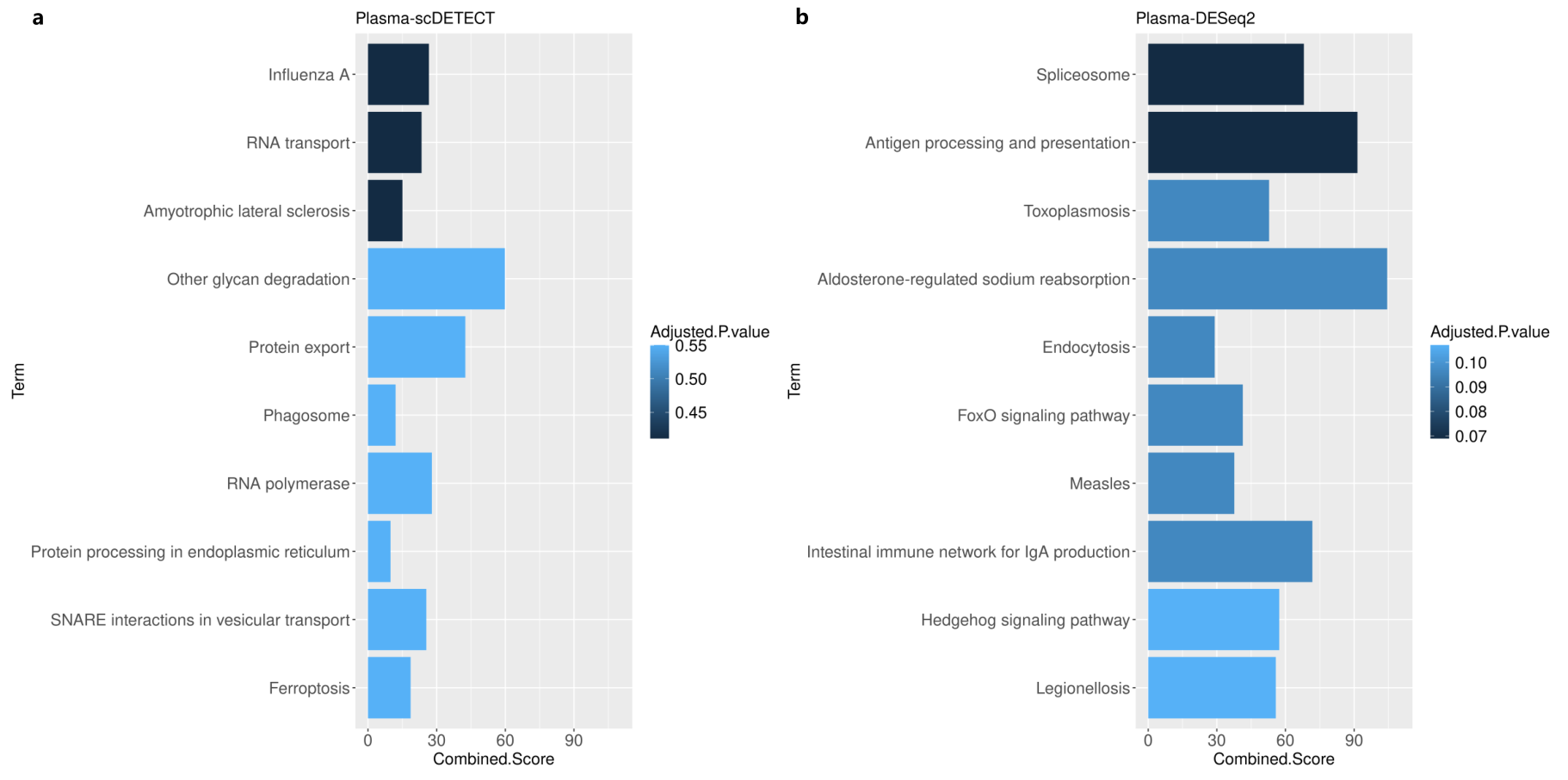
**

**Supplementary Figure S12. a.** KEGG pathways enriched by DE genes detected uniquely by scDETECT in Mega, but not by DESeq2. **b.** Pathways enriched by DE genes detected uniquely by DESeq2 in Mega, but not by scDETECT.

**
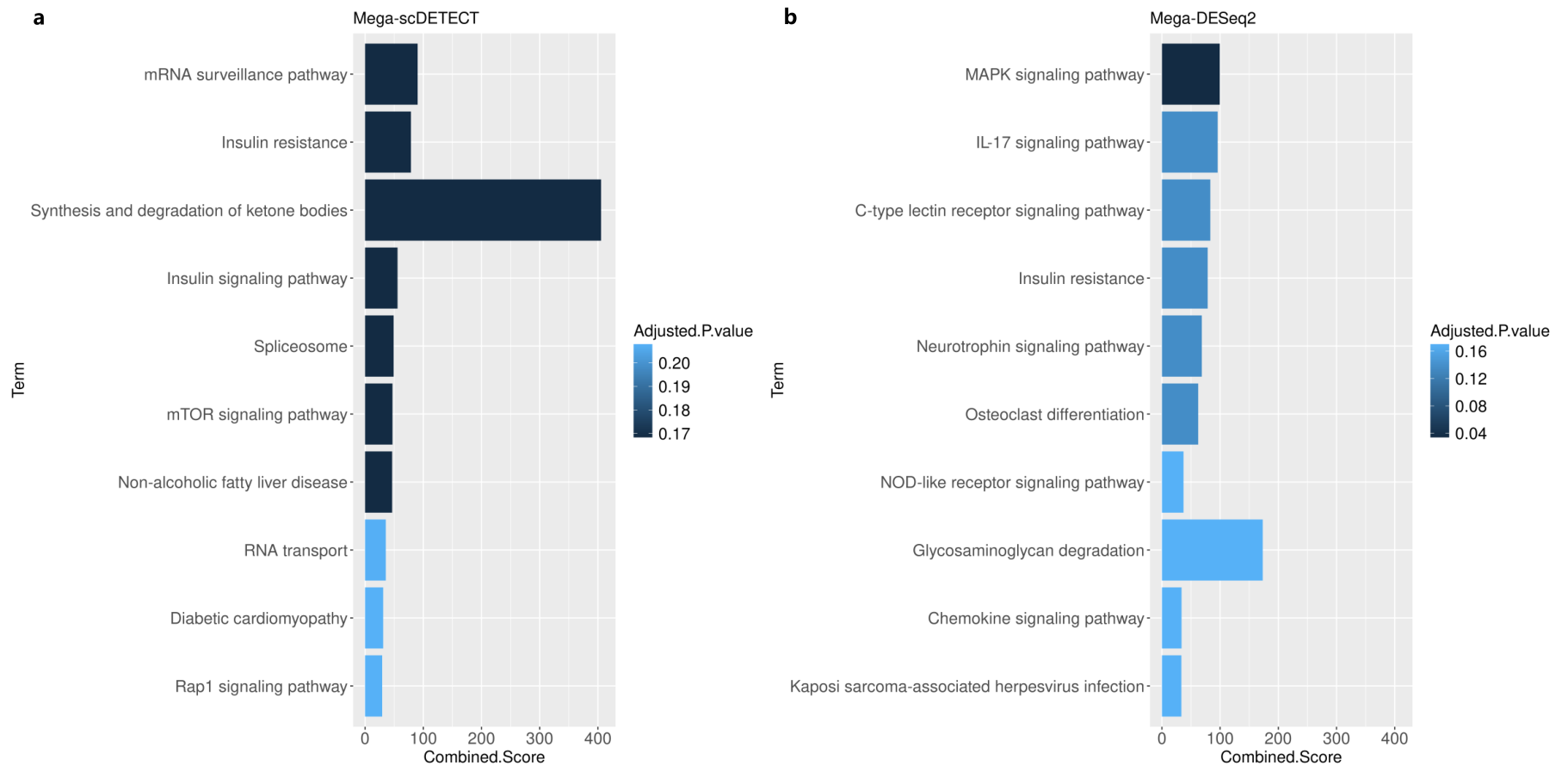
**

**Supplementary Figure S13.** Venn diagram showing overlap of DE genes detected by scDETECT, DESeq2, t-test and Seurat-MAST for all cell types in Lupus dataset.

**
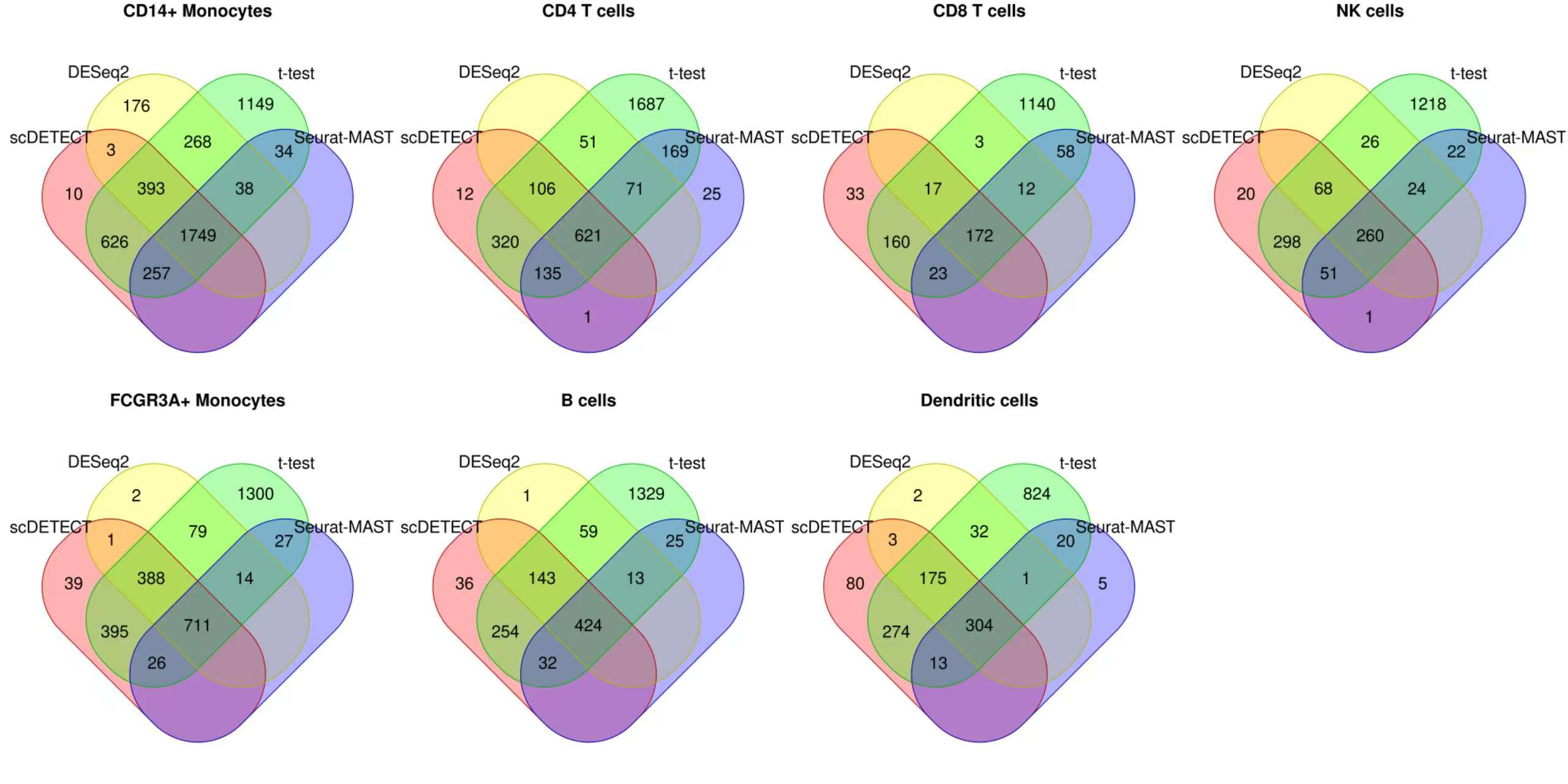
**

**Supplementary Figure S14.** Venn diagram showing overlap of top 500 DE genes detected by scDETECT, DESeq2, t-test and Seurat-MAST for all cell types in Lupus dataset.

**
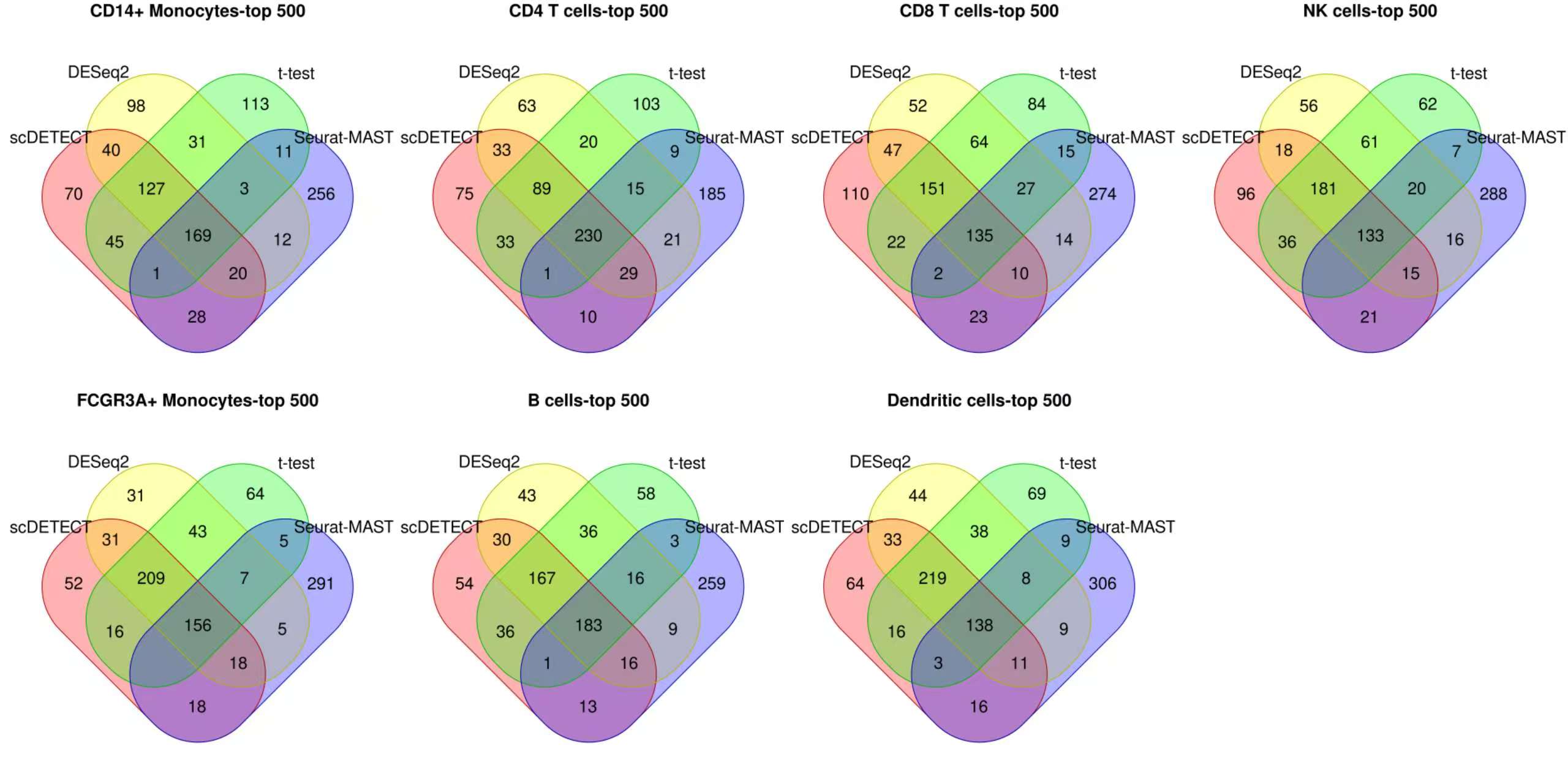
**

**Supplementary Figure S15.** Bar plots of the DE gene numbers of the four methods for all cell types in permutation data in Lupus dataset.

**
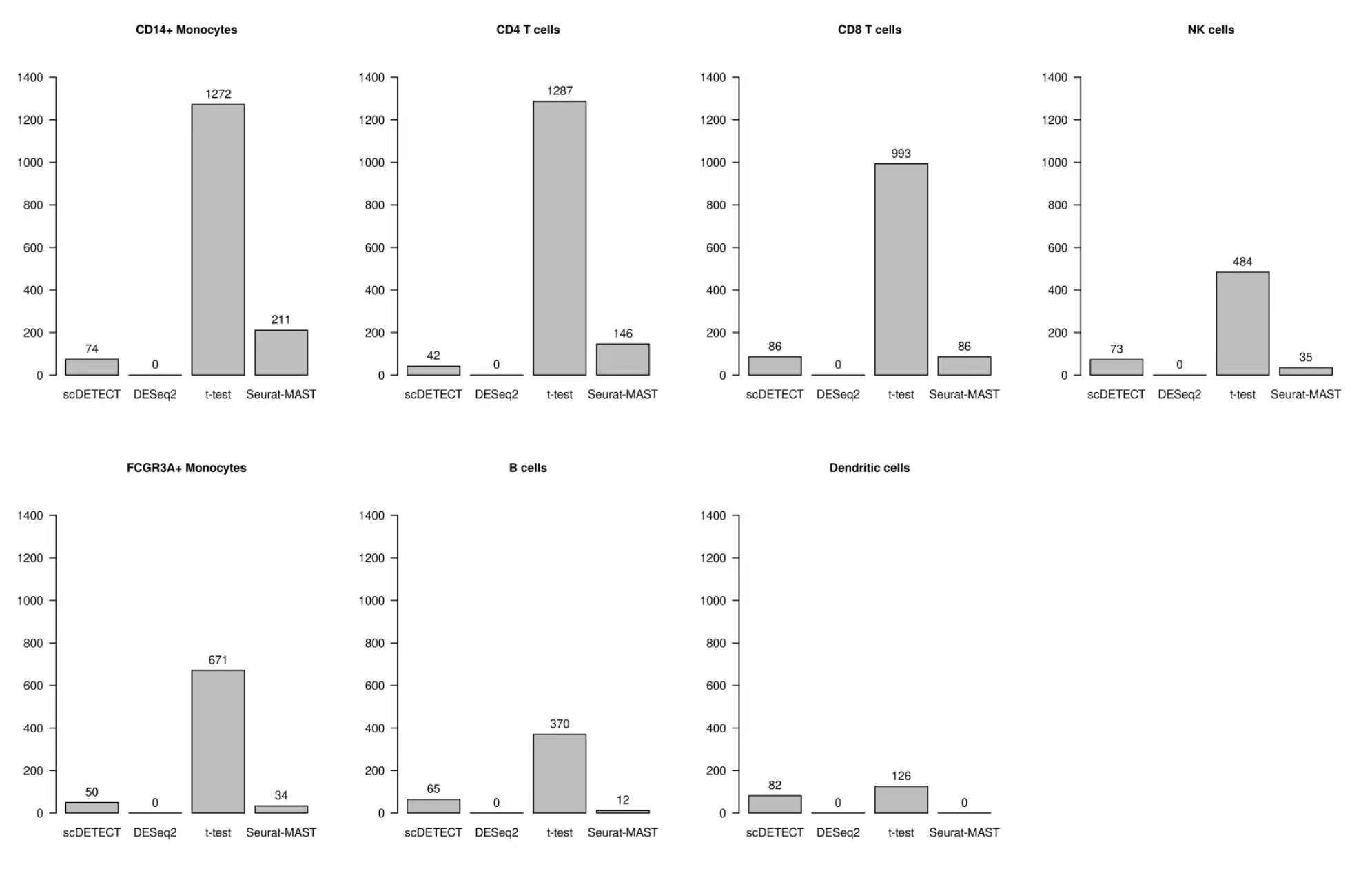
**

**Supplementary Figure S16.** Boxplot of overlap rates between DE genes detected from permutated datasets and the original dataset in Lupus dataset.

**
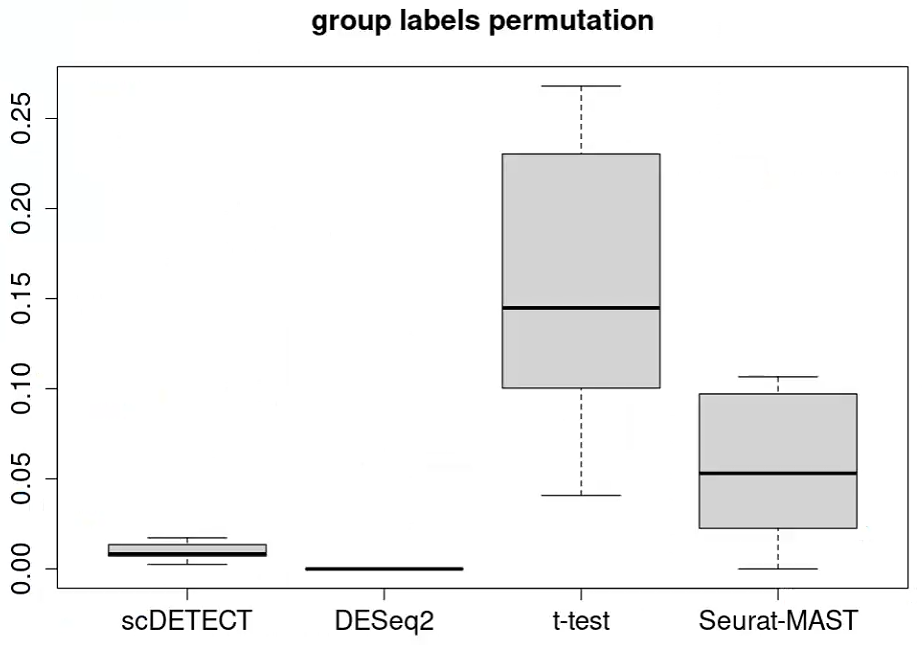
**

**Supplementary Figure S17.** Boxplots of overlap rates between DE genes detected from subsampled datasets and the original dataset: (a) 100 cells sampled from each cell type. (b) 2 individuals sampled from each group.


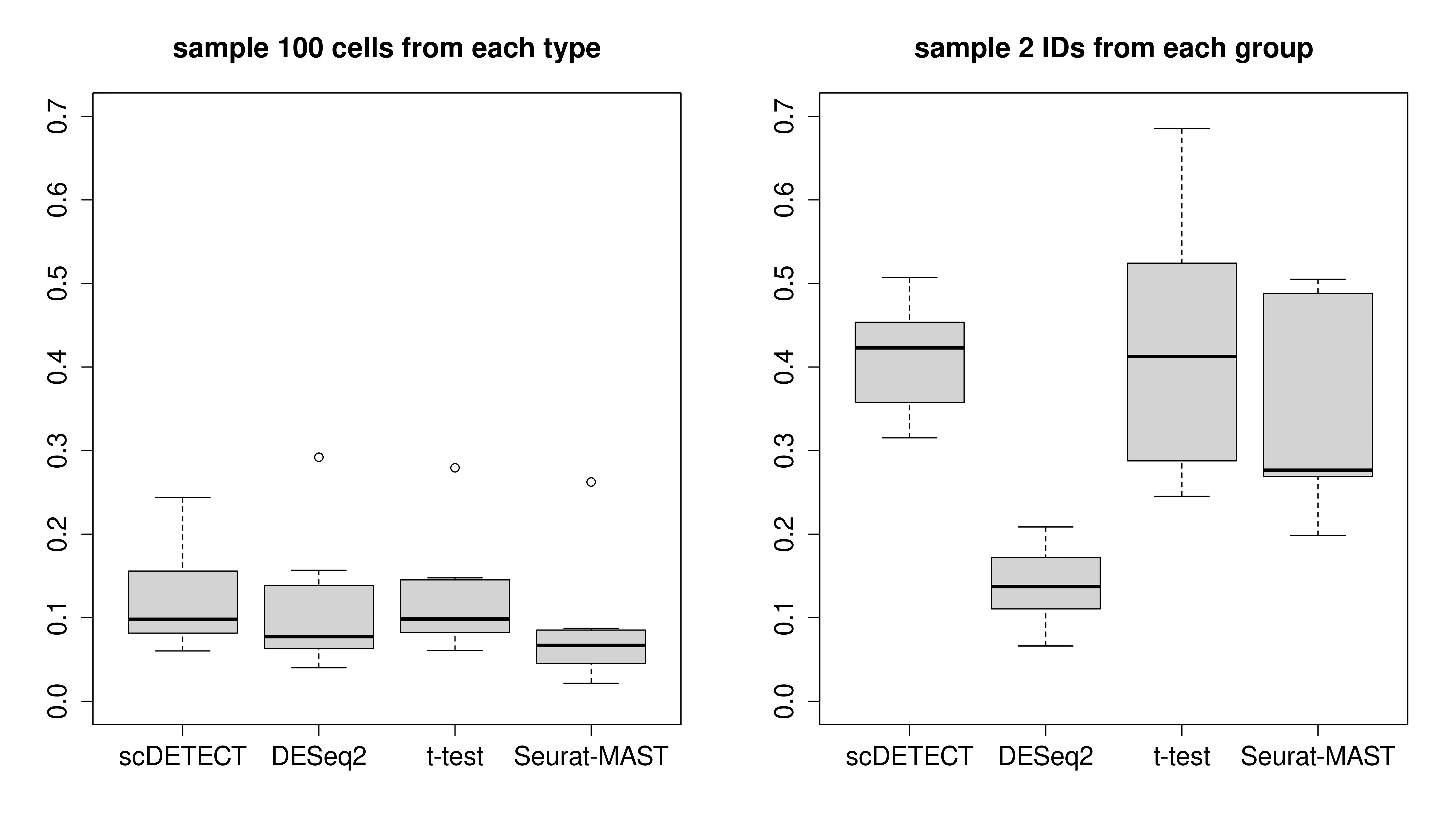


**Supplementary Table S1.** The RDR of the four methods for all the cell types.

| Cell types | scDETECT | DESeq2 | t-test | Seurat-MAST |
| --- | --- | --- | --- | --- |
| NK | 0.364 | 0.345 | 0.284 | 0.384 |
| B | 0.332 | 0.318 | 0.261 | 0.349 |
| CD4 | 0.472 | 0.464 | 0.402 | 0.368 |
| CD8 | 0.512 | 0.519 | 0.466 | 0.419 |
| Mono | 0.403 | 0.425 | 0.363 | 0.359 |
| DC | 0.216 | 0.201 | 0.186 | 0.352 |

**Supplementary Material 1.** The performance of BMHT is often dependent on the hierarchical tree structure, which always be estimated inaccurately. So we need to compare BMHT with different tree structures. We represent the results of one true tree structure (“tree 1”) and five mis-specified structures (“tree2”, “tree 3”, “tree 4”, “tree 5”, “tree 6”).


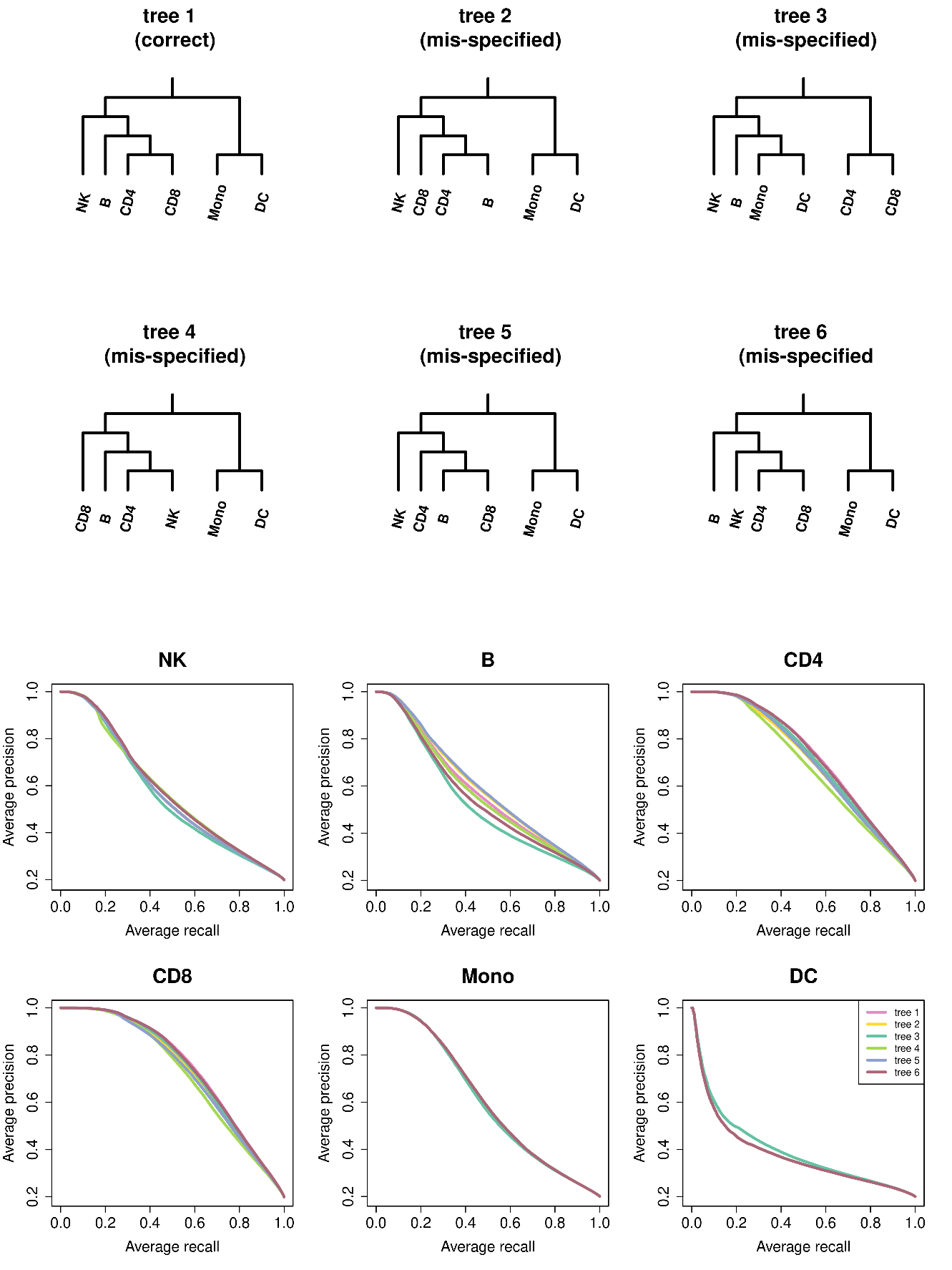


It is shown that BMHT is robust to mis-specified tree structures overall. But in some cases, the performance of BMHT with input of mis-specified tree structures still have small variations compared to true tree structure. When any cell type weakens correlation with cell types with large proportions, or strengthens correlation with cell types with small proportions, we can observe decrease of AUC-PR. For example, the AUC-PR for B with “tree 3” as well as “tree 6” has decreased compared to that with “tree 1”, so as CD4 with “tree 4”. This is because cell types with smaller proportions could not provide enough information as those with larger proportions. Besides, when the correlation levels of any cell type with any other cell types remain unchanged, we can get its PR curve that almost coincides with that of “tree 1” despite mis-clustering, such as NK with “tree 2” and “tree 5”. In addition, when any two cell types keep their strong enough correlation in the mis-specified tree, the change of AUC-PR is also small. For example, the PR curves for Mono and DC are basically coincident in all tree structures. CD4 and CD8 with “tree 6” also get almost the same result as that with “tree 1”.
